# Protein‐Based Encapsulation Strategies: Toward Micro‐ and Nanoscale Carriers with Increased Functionality

**DOI:** 10.1002/smsc.202100095

**Published:** 2022-01-18

**Authors:** Ricardo Ramos, Julien Bernard, François Ganachaud, Ali Miserez

**Affiliations:** ^1^ Université de Lyon INSA Lyon CNRS IMP 5223 Villeurbanne Cedex 69621 France; ^2^ INSA-Lyon, IMP Villeurbanne F-69621 France; ^3^ CNRS, UMR 5223 Ingénierie des Matériaux Polymères Villeurbanne F-69621 France; ^4^ Biological and Biomimetic Material Laboratory Center for Sustainable Materials (SusMat), School of Materials Science and Engineering Nanyang Technological University (NTU) 50 Nanyang Avenue Singapore 637 553 Singapore; ^5^ School of Biological Sciences NTU 59 Nanyang Drive Singapore 636921 Singapore

**Keywords:** encapsulation, membranes, microcapsules, nanocapsules, polypeptide-based simple coacervates, proteins

## Abstract

Proteins and peptides are attractive chemical building blocks to encapsulate and protect active substances thanks to their biocompatibility, biodegradability, low immunogenicity, and added functionality compared to synthetic polymers. This review provides a comprehensive overview of micro‐ and nanocapsules predominantly made of proteins—both natural and artificially produced—and peptides, detailing their different fabrication techniques and possible applications in various fields, including food technology and healthcare. Emphasis is given on the capability of proteins and peptides to assemble into capsular structures in the absence (e.g., protein cages and polypeptide‐based coacervates) or presence of a template, as well as on the physical nature of the carriers core, i.e., gaseous, liquid, or solid.

## Graphical abstract

An overview of micro‐ and nanocapsules made of proteins and peptides is presented, detailing their fabrication techniques and applications in various fields, including food technology and healthcare. Emphasis is given on the assembly of proteins and peptides into capsular structures in the absence or presence of a solid, liquid, or gaseous template.

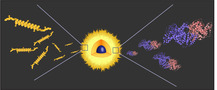

## 1 Introduction

The word “capsule” derives from the Latin word “capsula,” which can be translated to “small box” or “container.” In pharmacy, this term was adopted as early as the 19th century when physicians were facing a serious problem: they had medication that would improve the lives of patients, but due to their awful taste and texture, most people were refusing to go through with their treatment. In France, the oleoresin of copaiba (that possesses a nauseating taste) was prescribed to people suffering from venereal disease, whose incidence skyrocketed as a result of the Napoleonic wars and the associated social unrest. To solve this problem, in 1834 Mothes and Dublanc came up with the first capsules made of gelatin that masked the taste and smell of the encapsulated drugs, thus allowing a more pleasant consumption.^[^ ^1^ ^]^ Since then, encapsulation techniques have been widespread thanks to their numerous applications in protecting and delivering active ingredients such as drugs,^[^ ^2^ ^]^ cosmetics,^[^ ^3^ ^]^ fragrances,^[^ ^4^ ^]^ agricultural substances,^[^ ^5^ ^]^ and chemical reagents.^[^ ^6^ ^]^ In addition to the taste‐masking functions mentioned above, encapsulation strategies are useful to extend the shelf life of the (captured) substances, to protect them from the surrounding environment, and in some cases to transport and release them at specific sites.^[^ ^7^ ^]^

Capsules’ sizes can go from a few centimeters, like the ones developed by Mothes and Dublanc, to only a few tenths of nm depending on the desired application.^[^ ^8^ ^]^ When capsule size ranges from 0.1 to 100 µm, they are designated as microcapsules and if the size is in the 1−100 nm range, they are called nanocapsules (IUPAC definition).^[^ ^9^ ^]^ Microcapsules possess a larger inner volume than nanocapsules, which enables the loading of a higher amount of molecules of interest. In addition, thanks to their fragility, microcapsules can be easily broken by friction forces, which is highly desirable for applications such as cosmetics, flavor release, or textiles.^[^ 3, 10, 11 ^]^ In contrast, nanocapsules have very small sizes, can pass (biological) barriers, and deliver their cargos with enhanced precision compared to free therapeutic agents. This peculiarity has raised a lot of interest over the last two decades in drug delivery, and in situations where biological barriers pose a serious obstacle to the efficient delivery of active substances.^[^ 12, 13 ^]^ As an example, efficient drug delivery through the skin is a challenge for cosmetologists and dermatologists. For our own good, the skin is a strong barrier for outside penetration, especially thanks to the outmost layers of the epidermis being composed mainly of dead corneocytes surrounded by lipid layers. Studies have shown that even when using plasma treatment to render the skin more permeable, carriers larger than 700 nm are unable to penetrate it.^[^ ^14^ ^]^ A second example is the intravenous administration of drug‐filled carriers that requires sizes in the range of a few hundreds of nm. If the target is a tumor, microcarriers will have trouble passing the abnormal tumor vascular system composed of very small vessels where the blood travels slowly, whereas nanocapsules can more readily penetrate such an environment.^[^ ^15^ ^]^

All living systems use proteins to survive, from bacteria and viruses to the unicellular eukaryotes to plants and from vertebrates to higher mammals such as humans. Proteins make up 50% of the dry weight of cells and have unique roles in underpinning every reaction occurring in biological systems.^[^ 16, 17, 18 ^]^ Animal and plant proteins like albumin, hemoglobin (Hb), collagen, elastin, keratin, sericin, silk fibroin, soy protein, and many others are widely used in the biomedical, cosmetic, food, agriculture, and textile industries thanks to their biocompatibility, biodegradability, low inherent toxicity, and low toxicity of their degradation products. All of these characteristics make proteins very promising building blocks for the design of micro‐ and nanocapsules.^[^ ^19^ ^]^

To face the challenges associated with the controlled delivery of active ingredients for biomedical applications, encapsulation methodologies of high efficacy and reliability are necessary. Unfavorable solubility, undesirable toxicology, and nonspecific interactions characteristic of conventional release methods reinforce the necessity of investing in encapsulation research. Protein‐based micro‐ and nanocapsules offer several advantages over purely synthetic ones. For instance, the use of elastin‐based materials offer better chemical flexibility, biodegradability and temperature guided targeting and release mechanisms than synthetic polymers.^[^ ^20^ ^]^ In addition, protein modifications, coupling proteins with other functional molecules, and formulations of different types of proteins can enhance the versatility of this class of materials. In particular, the incorporation of specific functionalities at the surface of the capsules promotes efficient targeting, enhancement of transdermal transport properties, or increase of the effectiveness of the encapsulated (bio)active substances.^[^ ^21^ ^]^

In this review, the term “capsules” is loosely defined as micro‐ or nanoscale objects within which cargo molecules can be entrapped or encapsulated, and subsequently released by external stimuli. More specifically, we focus on proteins and peptides as the shell or encapsulant materials for these micro‐ and nano‐objects. The core can be: 1) gaseous, where typically gas bubbles are used as spherical templates for the proteins to form the shell; 2) liquid, where typically oil in water (o/w) or water in oil (w/o) emulsions are used as the template, or concentrated microdroplets are formed through a liquid–liquid phase separation (LLPS) process; and 3) solid, where the encapsulated molecules are directly used as the core or a solid nanoparticle is used to give shape to capsules before being degraded (generating hollow structures). Depending on the nature of the substance to be encapsulated, we present the different techniques used to generate the protein‐shelled microcapsules. We also discuss the different cross‐linking routes employed for stabilizing the capsules’ shells, including covalent bonding,^[^ ^22^ ^]^ the addition of external chemical stabilisers,^[^ ^21^ ^]^ or hydrogen bonding resulting from a change in protein conformation.^[^ ^23^ ^]^ Then, nanocapsules are discussed, either using a liquid or a solid core, or by direct self‐assembly of specific proteins into well‐controlled “cages.” Finally, artificial polypeptide‐based simple coacervates will be discussed because they have gained increased attention as promising encapsulation carriers for biomedical applications.

## 2 Routes to Protein Shell Microcapsules

### 2.1 Gas‐Filled Proteinaceous Microcapsules

Gas‐filled microcapsules find applications as contrast agents in 2D echocardiography, a technique that uses ultrasound reflection to image heart tissues in vivo. The microcapsules change the acoustic impedance of the blood flow, resulting in dramatically improved echo contrast in the surrounding tissues. Albunex and Optison are examples of commercially available sonographic contrast agents where air and octafluoropropane, respectively, are encapsulated in HSA membranes.^[^ ^24^ ^]^

#### 2.1.1 Sonochemical Technique

Sonochemistry is a scientific field that studies the chemical reactions induced by powerful ultrasound radiation (20 kHz–10 MHz). The underpinning phenomenon, called cavitation, refers to the formation, growth, and collapse of bubbles in a liquid. Bubbles collapse results in a massive increase of localized energy, producing high temperatures and pressures in the surrounding region of the liquid that is responsible for chemical excitation of any molecules that surround the bubble (acoustic cavitation).^[^ ^22^ ^]^ Acoustic cavitation in water generates H· and OH· radicals and these species generate H_2_O_2_ or HO_2_ in the presence of O_2_ (superoxide). In addition, ultrasonic irradiation of liquids is also known to create microscopic dispersions of gas in a liquid. When an aqueous solution of protein is sonicated, protein molecules attach at the air bubbles–water interface and can be cross‐linked by the formation of disulfide bonds between cysteine residues of the proteins. The cross‐linking reaction is triggered by the superoxide species mentioned above.^[^ ^22^ ^]^

**Figure 1:**
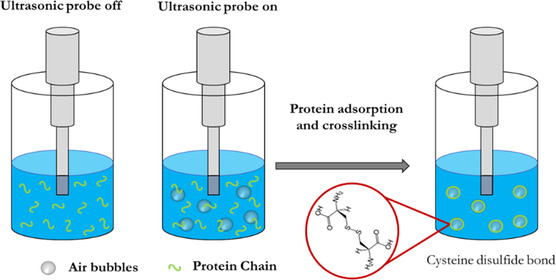
Fabrication of gas‐filled protein nanocapsules by the sonochemical technique. Redrawn based on the information presented in Ref. [25].

In 1991, Grinstaff and Suslick^[^ ^25^ ^]^ developed a procedure (see **Figure** **1**) to produce air–filled microcapsules by the sonochemical technique. They were able to synthesize 4 μm microcapsules of bovine serum albumin (BSA) and HSA by irradiating aqueous solutions of the proteins for 3 min at an acoustic power of 200 W cm^−2^ (20 kHz), pH 7, and an initial temperature of 50 °C. Twelve years later, Avivi and Gedanken^[^ ^26^ ^]^ extended the nature of proteins that could be used with the sonochemical technique to cysteine‐free proteins like streptavidin that possess a large amount of carboxyl groups on the side chains. The hypothesis was that at acidic pH (≤6) the hydrophobic interactions between protein chains would dominate and ensure the stabilization of the microcapsules. In their work, an aqueous solution of streptavidin was sonicated at an acoustic power of 150 W cm^−2^ (20 kHz) at pH 6 (adjusted with HCl) and at an initial temperature of 20 °C, to generate microcapsules with a diameter of around 5 μm that were stable for 1 month.

Air–filled microcapsules survive less than a minute in the blood circulation, especially if the patient is under oxygen therapy (because nitrogen diffuses from the microcapsule core to the blood), which limits their use as contrast agents. To solve this problem, insoluble gases like sulfur hexafluoride and perfluorocarbons like C_3_F_8_ (perflutren), C_4_F_10_ (perflubutane, PFB), C_5_F_12_ (perflenapent), and C_6_F_14_ (perflexane) were tested as air substitutes by several groups. One of the formulations that used perflubutane was reported as having a half‐life in mice of almost 10 min. To produce these microcapsules, a similar protocol has been used with the difference that protein aqueous solutions were purged with the gas that forms the core during the ultrasonication step.^[^ ^27^ ^]^

#### 2.1.2 Microfluidic Techniques

With advances in microfabrication technologies, new methods capable of producing gas‐filled microcapsules with extreme control over size and dispersity arose. Microfluidic platforms manipulate liquids and gases in channels that have a cross section between 10 and 100 μm. These devices are generally built from poly(dimethylsiloxane) (PDMS), a material extensively used for biological and water‐based applications, because it can easily be deposited on micropatterned molds and then strongly bonded to glass. Silicon wafers or glass can also be processed into microfluidic devices through photolithography and etching techniques.^[^ ^28^ ^]^ This technique allows precise control over size and dispersity of the microcapsules, which is of extreme importance for ultrasonic in vivo imaging, because the objects must have similar dimensions as red blood cells (6−8 μm) to safely pass through the microvasculature without diffusing across the endothelium.^[^ ^29^ ^]^

**Figure 2:**
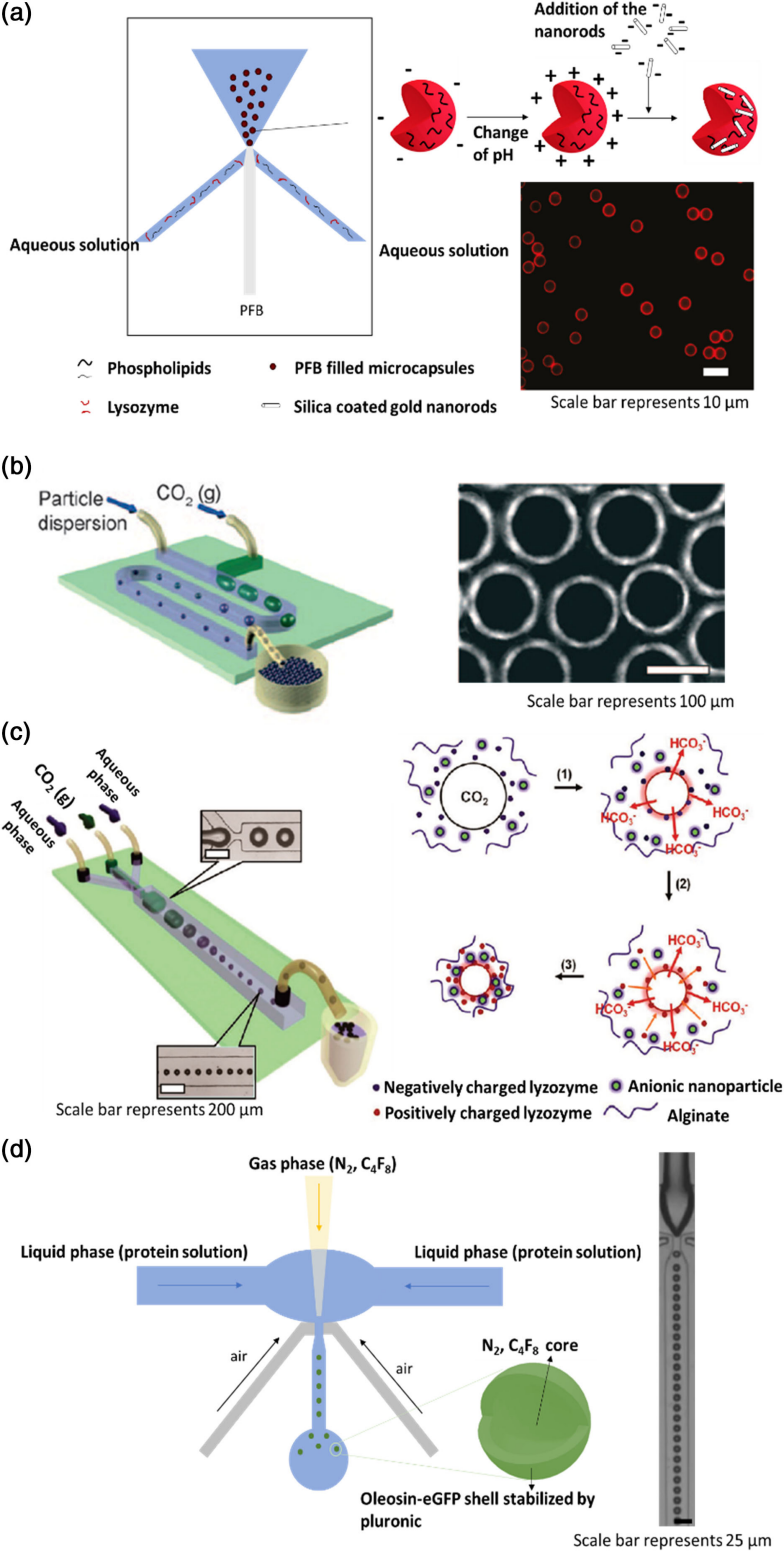
a) Microfluidic chip used to generate C_4_F_10_‐filled hybrid lysozyme/phospholipids microcapsules and strategy followed to electrostatically deposit the silica gold nanorods on the microcapsule surface. Fluorescence microscopy image of microcapsules loaded with silica coated quantum dots is shown on the right. Adapted with permission.^[^ ^30^ ^]^ Copyright 2010, American Chemical Society. b) Microfluidic device used for fabrication of armored microcapsules and fluorescence microscopy image of the resulting colloids. Reproduced with permission.^[^ ^31^ ^]^ Copyright 2009, Wiley‐VCH. c) Microfluidic device used to generate lysozyme/alginate‐covered microbubbles and scheme of the mechanism involved. Reproduced with permission.^[^ ^29^ ^]^ Copyright 2010, American Chemical Society. d) Microfluidic device used to produce recombinant oleosin shell microcapsules loaded with N_2_ or C_4_F_8_ gases together with an optical microscopy image of the capsules inside the channel of the device. Adapted with permission.^[^ ^32^ ^]^ Copyright 2014, American Chemical Society.

In 2010, Seo et al.^[^ ^30^ ^]^ developed a protocol using a PDMS microfluidic device to encapsulate PFB bubbles in a hybrid membrane composed of lysozyme and phospholipids, namely, 1,2‐dipalmitoyl‐*sn*‐glycero–3–phosphocholine and *N*‐(carbonyl‐methoxy‐polyethyleneglycol‐5000)‐1,2‐dipalmitoyl‐*sn*–glycero‐3‐phosphoethanolamine. The device, illustrated in **Figure** **2**a, contained three channels that intersected into one. In the center channel circulated the PFB, whereas the two side channels both pumped an aqueous solution containing the protein, the phospholipids, glycerol, and propylene glycol at pH 11.5, at which the lysozyme is barely negatively charged (its isoelectric point [IEP] is at pH 11.4). At the intersection of the channels, the aqueous phase surrounds the gas, shortly stopping its flow in a periodic manner and generating lysozyme shell microcapsules with diameters between 4 and 8 μm stabilized by the phospholipids. The authors took advantage of the IEP of lysozyme to promote hydrophobic interactions between the protein and the hydrophobic PFB. Posteriorly, the pH was changed to 7.4, causing the lysozyme to become positively charged and favoring the attachment of gold nanorods, CdSe/ZnS quantum dots, and Fe_3_O_4_ nanoparticles, all coated with a layer of negatively charged silica. This work intended to generate flexible gas‐filled microcapsules for imaging purposes.

In 2009, Park et al.^[^ ^31^ ^]^ reported on CO_2_ bubbles encapsulated with micrometer–sized beads of different materials, including BSA labeled with fluorescein isothiocyanate. A T‐junction microfluidic device (Figure 2b) was used, where CO_2_ gas and an aqueous solution of protein at pH 7 were introduced using a pressure regulator and a syringe pump, respectively. The dissolution of CO_2_ in water resulted in a local decrease in pH of the liquid adjacent to the bubble. The authors hypothesized that the decrease in pH would reach the IEP of BSA at 4.8, thus inducing the formation of 1−2 μm protein clusters that would precipitate on the bubbles surface. Microcapsules with a diameter of around 100 μm find application for the fabrication of thermal and acoustic insulators and lightweight materials with high structural stability. This work intended to be an alternative to current injection methods used to fabricate this kind of objects as it presents the advantage of producing samples with lower size dispersity.

One year later, the same authors adapted their protocol to fabricate CO_2_‐filled lysozyme/alginate‐based microcapsules containing anionically‐charged inorganic nanoparticles, such as Fe_3_O_4_, Au, or SiO_2_–encapsulated CdSe/ZnS nanoparticles, entrapped in the polymer membrane. As in their previous work, the authors relied on the local decrease in pH around the CO_2_ bubbles to positively charge the lysozyme and induce an electrostatically driven aggregation of the protein at the negatively charged bubble surface. The anionic nanoparticles and negatively charged alginate then deposited on the lysozyme‐coated bubbles, with the nanoparticles being predominantly located between the lysozyme and alginate shells. The team built a microfluidic device (Figure 2c) with three channels merging into one, where two of them were used to pump the liquid phase and the last one the gaseous phase. The liquid phase consisted of a mixture of lysozyme, alginate, and the anionic nanoparticles previously referred, and the gaseous phase contained CO_2_. The decrease in pH verified in the water close to the CO_2_ bubbles played an important role in the process, rendering the lysozyme positively charged which allowed its fixation on the negatively charged surface of the bubbles. The positively‐charged lysozyme also served as a substrate for the deposition of the negatively‐charged nanoparticles and alginate at the surface of the capsules through electrostatic interactions.^[^ ^29^ ^]^

In 2014, Angilè et al.^[^ ^32^ ^]^ reported microbubbles stabilized by a recombinant protein inspired by the amphiphilic oleosin protein from sunflower seeds. As oleosin in its native form has a low solubility in water and forms β‐sheets, the authors modified the oleosin gene by truncating a large portion of the hydrophobic domain, without changing the sequence otherwise, which resulted in a water‐soluble mutant protein that lost most of its secondary structure. In addition, five glycine residues were added into the remaining hydrophobic domain to increase the yield of protein expression, its stability, and solubility. They finally modified the oleosin gene by fusing it with an enhanced version of green fluorescent protein (eGFP) to confer emissive properties to the resulting protein.^[^ ^32^ ^]^ A PDMS microfluidic device, represented in Figure 2d, was used together with an air‐actuated valve to precisely control the size of the microcapsules. The liquid phase, supplied to the microfluidic device by a pump, consisted in the oleosin mutant protein and pluronic dispersants (namely, (PEO)_78_‐(PPO)_30_‐(PEO)_78_ or (PEO)_100_‐(PPO)_65_‐(PEO)_100_) diluted in phosphate buffer saline. The gas phase, i.e., pure nitrogen gas or octafluorocyclobutane (C_4_F_8_), was supplied to the device using a pressure regulator. This method yielded microcapsules with 10 μm in diameter, a narrow size distribution, and presenting green fluorescent properties.

### 2.2 Liquid Core and Protein Membrane Microcapsules

Protein microcapsules with liquid core are often generated from emulsion systems where a protein is deposited at the interface of the droplets and the solvent(s).^[^ ^33^ ^]^ According to IUPAC definition, emulsions are “fluid colloidal systems in which liquid droplets and/or liquid crystals are dispersed in liquid.”^[^ ^34^ ^]^ There are different types of emulsion depending on how the oil and water phases are organized. Simple emulsions are named w/o when the water phase is dispersed in a continuous oil phase and o/w when the contrary occurs. More complex emulsions designated “double emulsions” also exist, where an oil phase separates an external and internal water phase (w/o/w) or a water phase separates the oil phases (o/w/o).^[^ ^35^ ^]^

Different techniques of generating micrometer–sized emulsions are available, from ultrasonic emulsification to mechanical mixing to microfabrication. The following sections discuss how these techniques afford protein microcapsules and how they are sometimes paired with protein precipitation techniques to achieve the desired result.

#### 2.2.1 Sonochemical Emulsification Technique

This technique takes advantage of o/w emulsion. In contrast with the gas core microcapsules fabricated through the sonochemical technique discussed before, here a second liquid phase is introduced that will form an emulsion when using the ultrasonic probe. The core of the microcapsules is composed of the second phase that has to be immiscible with water. Again, protein cross‐linking occurs in the course of the sonication process through the formation of disulfide bonds triggered by the presence of superoxide species in the aqueous phase.

In 1990, Suslick and Grinstaff^[^ ^36^ ^]^ reported the preparation of oil–filled BSA–based microcapsules. To achieve this, o/w emulsions were prepared by emulsifying an organic solvent, i.e., *n*‐dodecane, *n*‐decane, *n*‐hexane, cyclohexane, or toluene, in an aqueous solution of BSA before the sonication step. The formation of protein microcapsules was confirmed by the addition of the water–insoluble 5,10,15,20–tetraphenylporphyrin (H_2_TPP), which exhibits a blue adsorption peak (418 nm in toluene).

**Figure 3:**
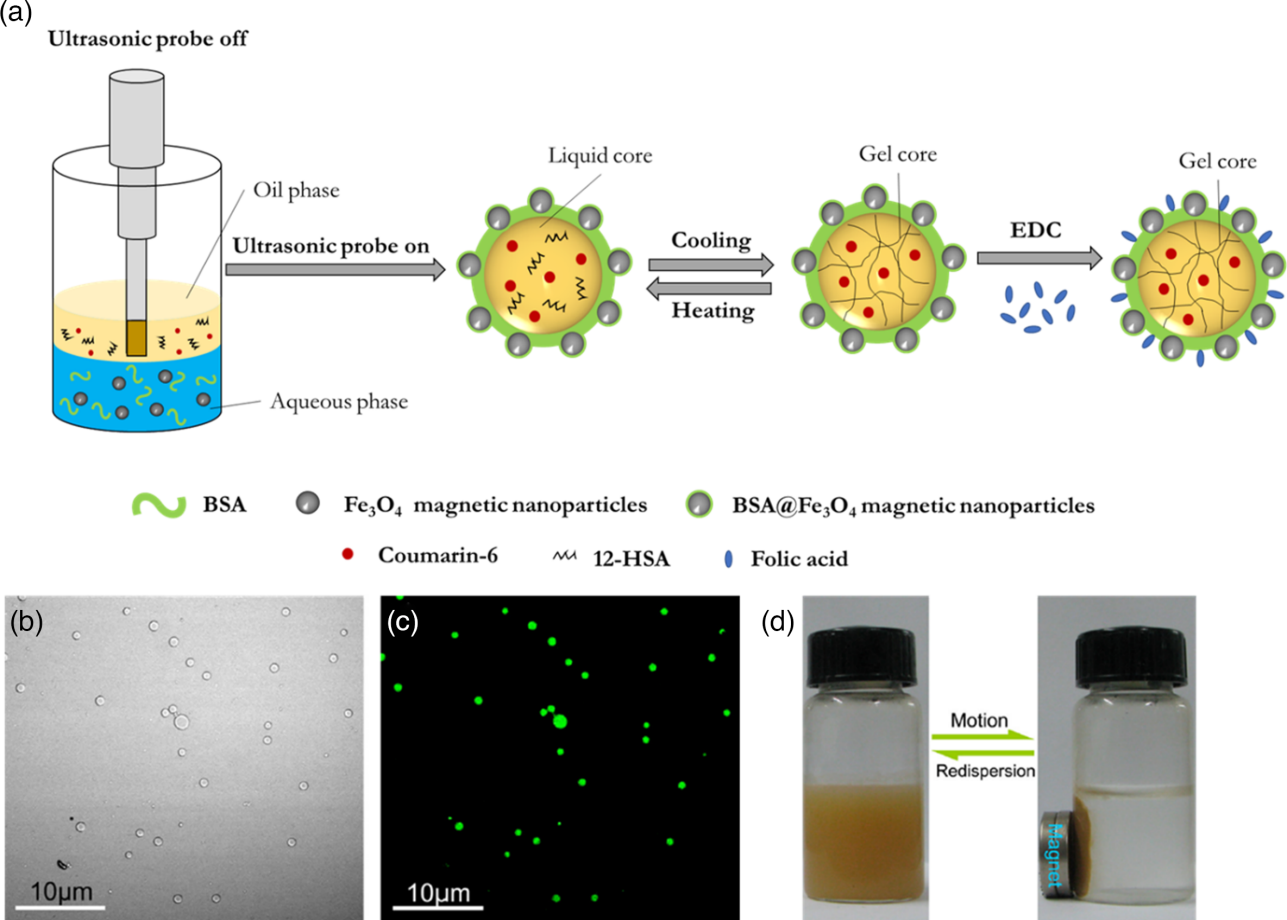
a) Fabrication of FA–BSA@Fe_3_O_4_ functionalized microcapsules through ultrasonic technique. b) Bright field and c) fluorescence confocal laser scanning microscopy images. d) Magnetic properties of the microcapsules. Adapted with permission.^[^ ^37^ ^]^ Copyright 2015, Elsevier B.V.

More recently, in 2015, Li et al.^[^ ^37^ ^]^ demonstrated the great potential of the ultrasonic technique to produce functional protein microcapsules for biomedical applications. In their work, Fe_3_O_4_ nanoparticles were functionalized with BSA chains to confer magnetic properties to the microcapsules. BSA molecules were immobilized on the surface of the nanoparticles through direct binding, using 1‐ethyl‐3‐(3‐dimethylaminepropyl) carbodiimide hydrochloride (EDC) and *n*‐hydroxysuccinimide (NHS) to activate the carboxyl groups of the protein. A two‐phase solution was produced by adding soybean oil containing 12‐hydroxyoctadecanoic acid (12–HSA) to an aqueous solution containing BSA@Fe_3_O_4_ and BSA at a 1:80 ratio. The mixture was sonicated for 6 min at a power of 350 W cm^−2^ at 40 °C (see **Figure** **3**). 12–HSA was added to tune the fusion temperature of the oil phase so that it is a thickened gel below 37 °C and a liquid above. To give a dual targeting ability to the microcapsules (leveraging on the magnetic targeting from the Fe_3_O_4_ nanoparticles), folic acid was further attached onto the BSA‐based shell of the microcapsules using EDC (FA‐BSA@Fe_3_O_4_). Folate receptors are known to be overexpressed by some tumors.

The resulting microcapsules had diameters between 0.2 and 1.9 μm and exhibited magnetic properties, aggregating on the vial wall when exposed to an external magnetic field. A hydrophobic fluorescence dye, coumarin–6, was finally encapsulated in the oily core of the microcapsules by dispersing it in the thermosensitive oily mixture before sonication to study the release profiles of the capsules. A small difference was observed in the release profiles at 35 and 40 °C, over a period of 5 days, with more coumarin being released at the higher temperature when the core was liquid compared to the lower one where the core was gelified (24 vs 16% of the total encapsulated dye molecules, respectively).^[^ ^37^ ^]^

#### 2.2.2 Protein Coacervation Emulsification Techniques

Coacervation consists in a phase separation where fully solvated macromolecules are desolvated upon addition of a salt, an electrolyte compound, a nonsolvent, and/or after a temperature or pH change. The phase rich in polymer is named coacervate and the equilibrium phase is the name given to the other phase, mainly composed of solvent. The coacervation can be defined as simple, when only one macromolecule takes part to the precipitation process, or complex, when two oppositely‐charged polymers interact in a solution.^[^ ^33^ ^]^ When used in combination with emulsification techniques to conceive precisely defined templates, coacervation of proteins can lead to the formation of microcapsules (vide infra). This section focuses on coacervate microcapsules made of proteins extracted from natural sources. Coacervate micro‐ and nanodroplets built from synthetic short and longer polypeptides are presented in Section 4.

In 2002, Mauguet et al.^[^ ^38^ ^]^ described the preparation of microcapsules from hexadecane and gliadin, a cereal protein entering in the composition of gluten. The microcapsules were generated by emulsifying hexadecane in an acetic acid aqueous solution, within which the protein was dissolved by mechanical stirring, followed by the addition of NaCl. Using this protocol, microcapsules with a mean diameter around 170 μm were fabricated at a protein concentration of 5 mg mL^−1^ and a salt content of 1.2 mg mL^−1^. Mauguet's team also explored how protein or salt concentration and the rate of salt addition affected the aggregation of the microcapsules. They found that high concentrations of gliadin (11−13 mg mL^−1^) resulted in microcapsule aggregation even when low amounts of coacervation agent were used (less than 1 mg mL^−1^). At low concentrations of protein (below 5 mg mL^−1^), higher concentrations of salt (above 8 mg mL^−1^) were required to coacervate the gliadin. The rate of salt addition also had a strong impact on the aggregation process because gliadin is particularly sensitive to ionic strength variations. A slow addition of salt proved to be essential to prevent aggregation. The cross‐linking of the protein shell was subsequently performed at 40 °C and pH 8.9 to promote deprotonation of the amine groups and reaction with glutaraldehyde (GA).

In 2004, Lazko et al.^[^ ^39^ ^]^ reported the fabrication of soy glycinin microcapsules filled with hexadecane by simple coacervation. To generate the microcapsules, hexadecane was added to an aqueous solution of glycinin and the medium was acidified by addition of HCl to reach pH 2. After emulsification of the hexadecane by magnetically stirring the solution at 55 °C, precipitation of the protein was induced by pH shifting to 5 using an aqueous solution of NaOH. After cooling down the mixture to 25 °C, the addition of an aqueous solution containing GA induced cross‐linking of the precipitated protein around the oil droplets. Microcapsules with a diameter between 100 and 200 μm were obtained by varying the different parameters of the reaction.

Ionic interactions between two or more oppositely‐charged macromolecules lead to phase separation by complex coacervation. Proteins are commonly coacervated together with polysaccharides, thus forming hybrid coacervates.^[^ ^40^ ^]^ The first microcapsules fabricated by this method date back to 1957 when Green encapsulated dyes in gelatin/acacia gum microcapsules in an effort to develop carbonless copy paper.^[^ ^41^ ^]^ Another notable example of microcapsules generated by complex coacervation is the work of Fergason, patented in 1984, where nematic liquid crystals were encapsulated in hybrid gelatin/acacia gum microcapsules and used in visual display devices.^[^ ^42^ ^]^

**Figure 4:**
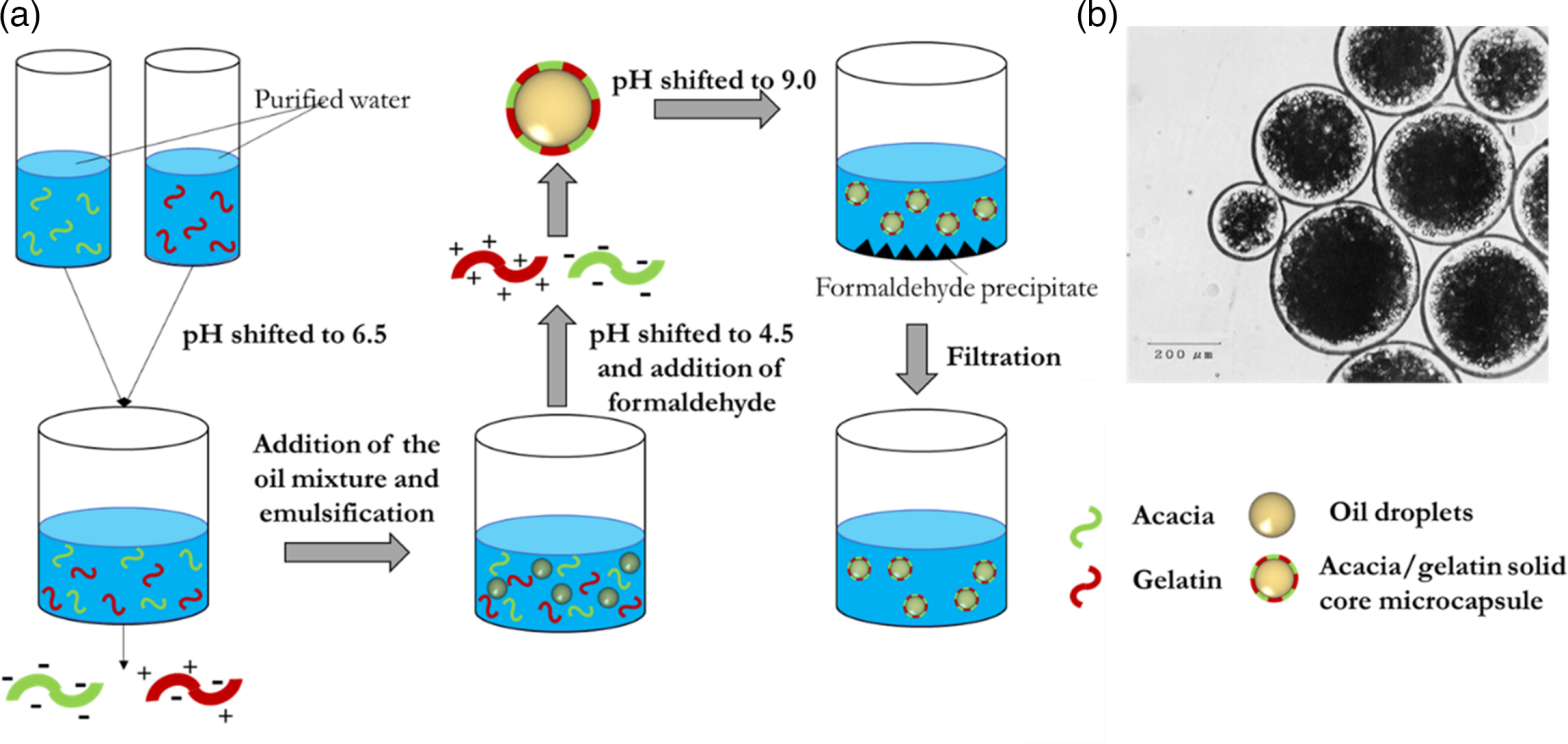
a) Scheme of the complex coacervation‐driven synthesis of acacia/gelatin microcapsules with liquid core. Illustration is based on the information presented in Ref. [43b]. b) Optical microscopy image of the gelatin/acacia gum microcapsules containing probucol. Reproduced with permission.^[44]^ Copyright 1993, Springer Nature.

Most of the literature on microcapsules generated by complex coacervation of proteins was restricted to patents until 1970. Two of the first studies were published in 1964 and 1967 by Luzzi and Gerraughty.^[^ ^43^ ^]^ These authors explored the complex coacervation of acacia gum and gelatin to encapsulate oil droplets previously emulsified. Both macromolecules are good candidates for complex coacervation thanks to their IEP difference (gelatin pH 8 and acacia gum pH 2), which allows them to coexist in the same solution without interacting electrostatically at pHs close to or higher than 8. This facilitates the control of coacervation because a slight shift in pH of the solution triggers the process. To prepare microcapsules, Luzzi and Gerraughty first dissolved acacia gum and gelatin separately in purified water. Before mixing the two solutions, the pH of the solutions was adjusted to 6.5. As acacia gum is negatively charged at pH 6.5 and gelatin still has enough negative charges to prevent interactions with acacia, this pH prevents electrostatic interactions between the proteins. Mixtures of different compositions of petrolatum and coconut oil were emulsified by adding the mixture of oils to the polymer aqueous solution and mixing them with a hand homogenizer. The pH was then adjusted to 4.5 by addition of diluted HCl while stirring to positively charge the gelatin and start the coacervation process (acacia gum is negatively charged at this pH). To cross‐link the microcapsules, formaldehyde was then added and the temperature was reduced to 10 °C. The formaldehyde denatured the gelatin–acacia gum complexes and permanently stabilized the capsules. Finally, the pH was adjusted to 9 and the sample was filtrated to recover the coacervate and eliminate excess formaldehyde. **Figure** **4**a summarizes microcapsule fabrication by acacia gum/gelatin complex coacervation emulsion technique. Microscopy images shown in the study demonstrated the presence of microcapsules but the average size was not mentioned.

Later, Jizomoto et al. developed a similar protocol to generate gelatin/acacia gum microcapsules with a core made of oil and lipophilic drugs. The oil phase used to generate the emulsion was chosen as a mixture of glyceryl tricaprylate/tricaprate (ODO) and probucol, a drug which used to be prescribed to treat coronary artery disorder disease.^[^ ^44^ ^]^ The protocol started by the dissolution of probucol in ODO oil mixture, followed by the addition of the resulting solution to purified water, warmed to 50 °C. The o/w emulsion was formed by mechanical mixing using a homogenizer. An aqueous solution of acacia gum was added to the emulsion and the coacervation process was initiated by adjusting the pH to 4 (by addition of an aqueous solution of diluted acetic acid). This process resulted in spherical microcapsules (shown in Figure 4b) with a size ranging between 130 and 200 μm and a drug content of 78.1 mg g^−1^ of microcapsule.^[^ ^44^ ^]^

A more recent study on oil‐filled microcapsules produced by complex coacervation of a protein and a polysaccharide was published by Deveci and Basal.^[^ ^45^ ^]^ In their work, silk fibroin and chitosan were coacervated together to form *n*–eicosane‐filled microcapsules. The silk fibroin solution was heated to 50 °C followed by the addition of different amounts of *n*‐eicosane. The authors aimed to produce microcapsules that could be used in thermoregulated textiles, where phase‐changing materials (like *n*–eicosane) absorb, store, and release heat. The o/w emulsion was formed by mechanical stirring. Sorbitan laurate (Span‐20), a surfactant, was added to stabilize the system. Considering the IEP of silk fibroin (pH 4.2) and the pKa of chitosan (pH 6.5), the pH of the mixture was adjusted to 5.2. The final step consisted in the addition of GA to cross‐link the microcapsule shells. Deveci and Basal explored how silk fibroin/chitosan ratio, oil content, and cross‐linking density influenced microcapsule size and encapsulation efficiency. The average microcapsules presented a mean diameter of 23 μm and an encapsulation efficiency of 64%. Advantageously, these parameters could be tuned. Higher silk fibroin/chitosan ratios and higher fractions of *n*‐eicosane resulted in higher encapsulation efficiencies up to a maximum of 74%. The microcapsules’ size could be tuned from 8 to 38 μm and followed a similar tendency, with bigger sizes being observed for higher silk fibroin/chitosan ratios and higher percentages of *n*‐eicosane.

Several other studies on microcapsules fabricated using complex coacervation of proteins have been reported over the years (some examples of which are compiled in **Table** **1**). Most of these studies mixed polysaccharides with proteins to generate the objects. Such approaches have been reviewed in detail elsewhere.^[^ ^46^ ^]^

**Table 1:** Microcapsules produced by complex coacervation. SPI is short for soy protein isolate

Biopolymers		Ratio	pH	Core	Size [μm]	References
SPI	Acacia gum	1:1	4.4	Sweet orange oil	7.6	[117]
SPI	Pectin	1:1	4.4	Soy oil and casein hydrosylate	16–24	[118]
Chitosan	Acacia gum	1:4	3.6	Miglyol	1–2	[119]
Whey protein	Acacia gum	2:1	4.0	Orange oil flavor and sunflower oil	50–150	[120]
Gelatin	Acacia gum	1:1	4.0	Sunflower oil and aspartame	84–102	[121]

#### 2.2.3 Microfluidic Techniques

Microfluidic devices can be used to fabricate liquid‐filled microcapsules. In 2016, Schloss et al.^[^ ^47^ ^]^ developed a protocol to produce microcapsules with modified bacterial biofilm surface layer protein (BSlA) shell. BSlA is a low molecular weight (19.1 kDa) amphipathic protein with a large hydrophilic domain and a narrow hydrophobic cap. In water, the hydrophobic side chains are buried in a random coil conformation such that the protein forms β–sheets at amphiphilic interfaces. This metamorphism plays a role not only in protein assembly at oil/water interfaces, but also in preventing aggregation in aqueous suspensions, which are characteristics that are highly desirable for microcapsule fabrication.

BSlA was obtained by cloning its gene into the pGEX–6 P–1 vector using specific restriction sites to give the desired fusion protein expressed in *Escherichia coli.* The microcapsules were then engineered using a T‐junction microfluid device with three channels where an aqueous solution of protein circulated in two channels perpendicular to the third, where mineral oil was pumped through. To facilitate the functionalization of BSlA with other proteins, Schloss’ team genetically engineered constructs that fused a 13‐residue tag to one of the terminals of BSlA. This tag contained a lysine side chain that forms a covalent bond with an aspartic acid side chain of a tag engineered into another protein (SpyCatcher‐SpyTag system). As a proof of concept, the tagged BSlA was covalently grafted to a tagged GFP, conferring fluorescent properties to the microcapsules.

**Figure 5:**
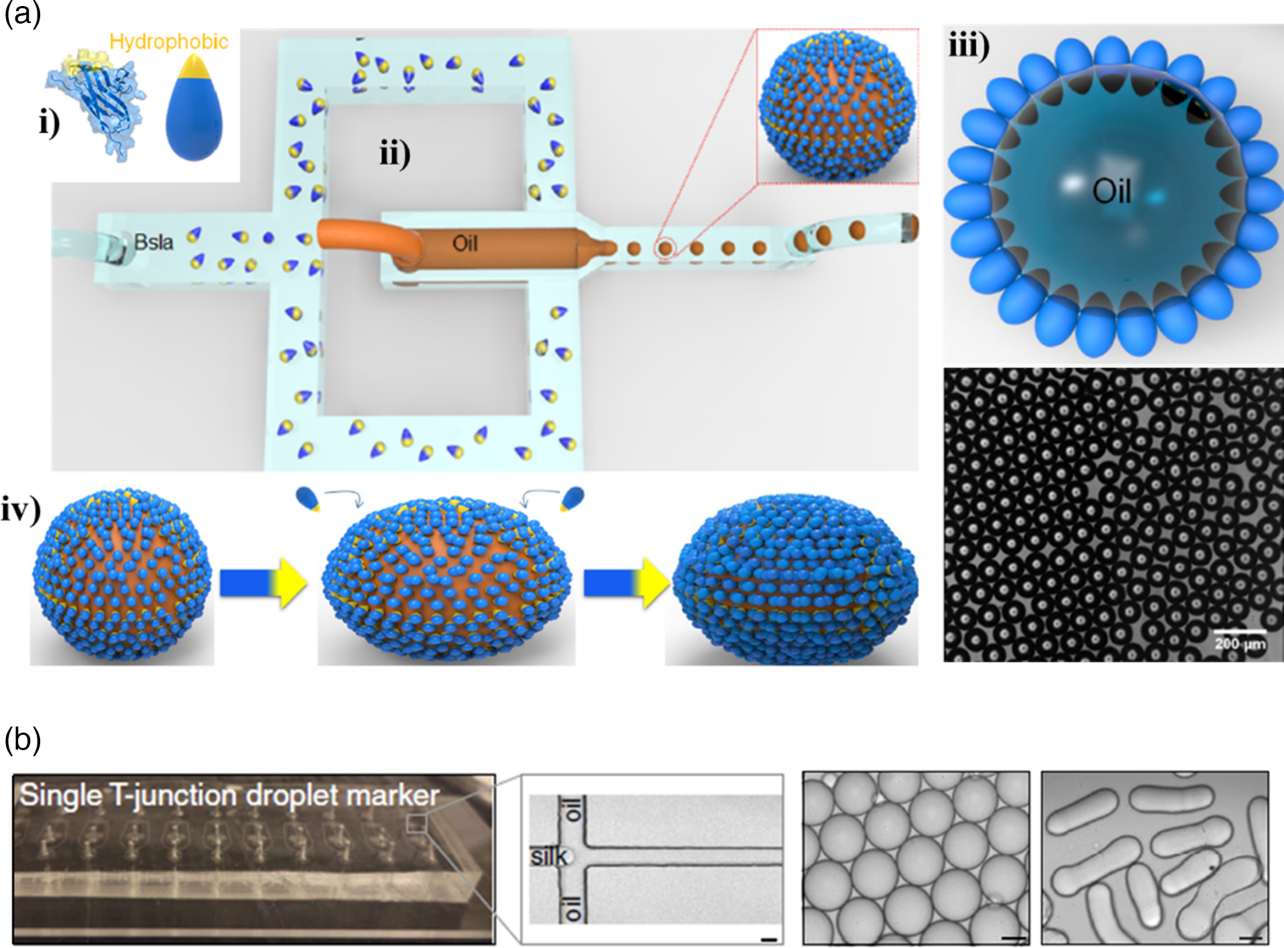
a) Schematic of the i) BSlA structure in ribbon and cartoon form, ii) microfluidic device used to fabricate the microcapsules, iii) cross section of a microcapsule showing the packing assembly of BSlA at oil–water interfaces together with an optical microscopy image of the BSlA microcapsules, iv) representation of microcapsule deformation due to adsorption of free BSlA onto the excess interface exposed by droplet elongation. Reproduced with permission.^[^ ^48^ ^]^ Copyright 2017, American Chemical Society. b) Microfluidic device used to fabricate silk fibroin micrococoons (scale bar represents 20 μm) and optical microscopy images of spherical and cylindrical micrococoons (scale bars represents 10 μm). Reproduced under the terms of the CC‐BY 4.0 license.^[49a]^ Copyright 2017, The Authors, published by Springer Nature.

One year later, the same group described the production of w/o and o/w emulsions stabilized by BSlA using a similar microfluidic device.^[^ ^48^ ^]^ This time no tag was used because the goal of the study was to characterize the difference in organization of the protein when oil or water was used as the continuous phase. **Figure** **5**a shows how BSlA self‐organizes at the surface of oil and water droplets. The researchers found that due to its interfacial organization, the BSlA protein formed elastic films at amphiphilic interfaces, which resulted in capsules that can be irreversibly deformed when subjected to an external force. Stable, monodispersed oil‐filled microcapsules with a mean diameter of 100 μm could be readily produced, whereas water‐filled microcapsules presented arrested coalescence.

In the same year, Shimanovich et al.^[49a]^ described the production of native silk fibroin micrococoons with controllable geometry and β‐sheet content by taking advantage of w/o emulsions. The cocoons were fabricated using a T‐junction microfluidic device by co‐flowing an immiscible fluorinated oil phase (fluorinert FC‐70) from both sides of a stream of native silk protein dissolved in water at pH 7 that circulated in a central microchannel. By tuning the concentration of native silk fibroin and flow rate of the two phases, the authors were able to fabricate micrococoons with spherical or cylindrical shape (Figure 5b). The tendency of the stream breaking into droplets led to the formation of a water‐in‐oil emulsion at the T‐junction, and shearing the emulsion converted the native silk fibroin into an aggregated state at the aqueous/oil interface where the shear is greatest. The micrococoons proved capable of preserving the activity of sensitive cargo proteins, such as antibodies (single‐chain Fv‐binding domain specific for the protein huntingtin, C4scFv, and two single‐chain Fv domains specific for α‐synuclein, NbSyn86, and NbSyn87) that can aggregate and lose function under conditions of bulk storage. These antibodies were encapsulated by incorporating them in the aqueous phase together with the native silk fibroin. More recently, this team used the same setup to generate microcapsules from a 13 amino acid‐long peptide (called “KD”) derived from human semenogelin I protein.^[49b]^ The microcapsules, which consisted of smaller β‐sheet fibrillar structures, were induced by a pH shift from their soluble state (pH < 6) to pH 8 and could be disassembled by reverting the pH back to 6. Furthermore, the size of the microcapsules could be scaled down to the submicrometer level using the nanofluidic system described above, and the authors were also able to encapsulate C4scFv.

#### 2.2.4 Spray Drying Technique

**Figure 6:**
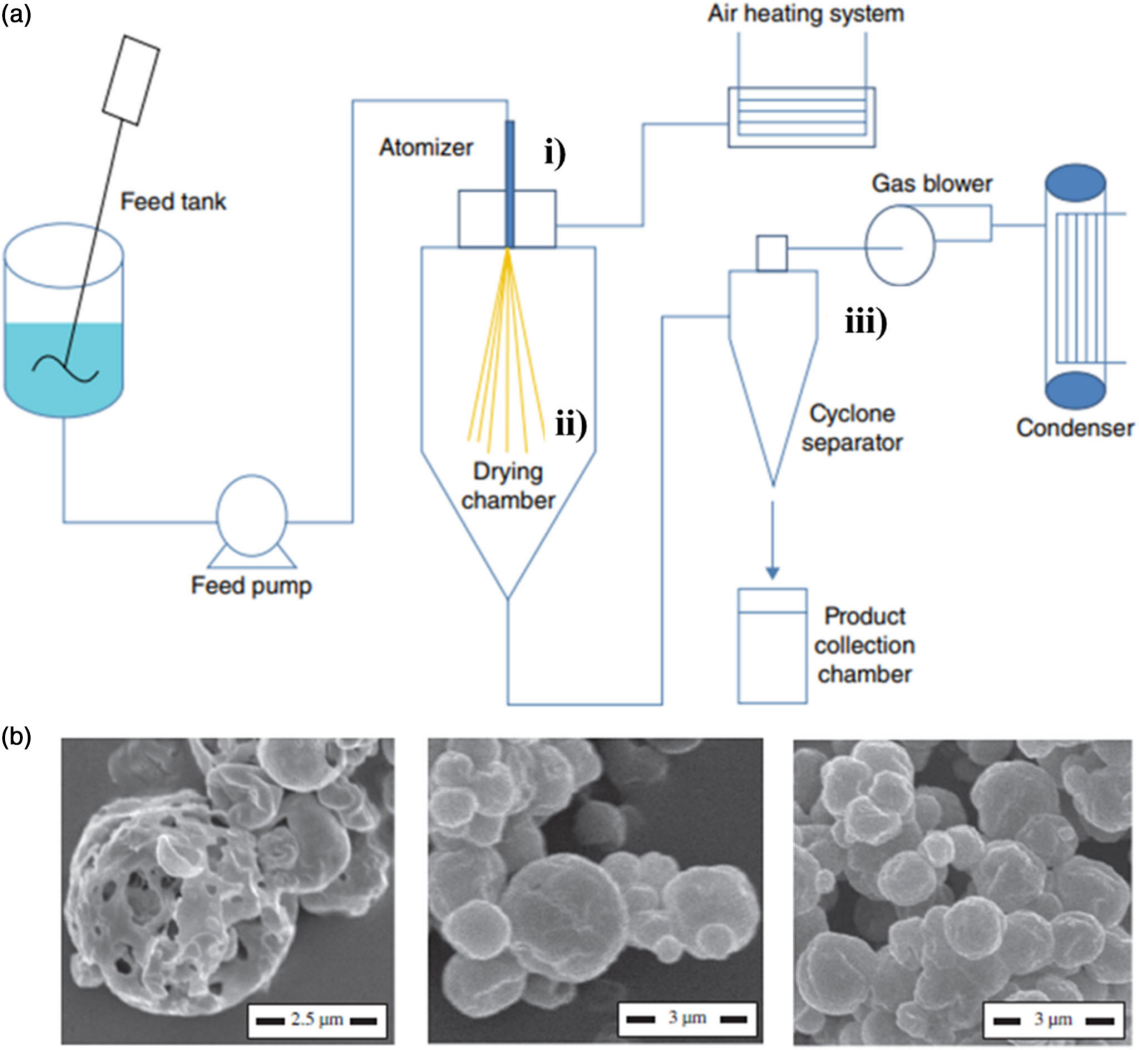
a) Steps of spray drying: i) atomization of the liquid into droplets; ii) mixing of the droplets with a heated gas stream; iii) separation of the powder from the gas stream and its recollection. Reproduced with permission.^[^ ^50^ ^]^ Copyright 2015, John Wiley & Sons. b) SEM images of microcapsules with a shell made of hordein (left), a 1:1 ratio of glutelin and hordein (center), and glutelin (right). Reproduced with permission.^[^ ^52^ ^]^ Copyright 2011, Elsevier Ltd.

Spray drying was invented in the 1870s in the USA. Due to the low technological development at the time, it was not implemented at industrial scale until the second half of the 20^th^ century. The first major application of this technique was the production of milk powder (by drying milk), whey, and baby formulas, which are still the biggest commercial outcomes. Encapsulation by spray drying has been used by the food industry since the late 1950s to provide flavor oils with protection against oxidation and degradation, and to convert liquid phases into solid ones. Its continuous nature and adaptability to industrialization makes spray drying the most commonly used technique by the food industry to encapsulate oils.^[^ ^50^ ^]^ Encapsulation by spray drying consists in a dehydration process that generates a continuous matrix around a core. The process starts by preparing an initial solution containing the wall and core materials and spraying it into a stream of hot air, which evaporates the solvent, resulting in a powder of microcapsules. There are three fundamental steps involved in this technique: 1) atomization of the liquid into droplets; 2) mixing of the droplets with a heated gas stream; and 3) separation of the powder from the gas stream and recollection (steps presented in **Figure** **6**a).^[^ ^50^ ^]^

Protein shell microcapsules fabricated through spray drying have been studied as protective containers to prevent degradation of vegetable or fish oils. In 1996, Kim et al.^[^ ^51^ ^]^ reported the fabrication of microcapsules composed of a shell made of whey and soy protein isolates and an orange oil's core (extracted from the rind of orange) by spray drying of an aqueous solution containing the oil and the proteins. This work intended to find a replacement for carbohydrates (such as starch and gums) like acacia gum that were commonly used as wall materials. The authors were able to fabricate microcapsules using the two proteins and found that even though their size was similar (around 40 μm), the soy protein isolate microcapsules presented a higher encapsulation efficiency (85.7% against 72.7%).

Wang et al. published a study in 2011^[^ ^52^ ^]^ on a spray drying protocol for the fabrication of fish oil‐filled microcapsules with shell made of hordein and/or glutelin. These proteins, extracted from barley grains and its by‐products, can be easily emulsified or processed into films and can stabilize emulsions by forming microcapsules. Using a wall material that generates chemically‐active capsule surfaces, the goal of the work was to encapsulate fish oil to hide its unpleasant taste and favor its digestion with no negative effects on health. To this end, Wang's group adapted the spray drying protocol commonly used for other proteins to generate 1–5 μm microcapsules with a shell made of one of these two proteins or of mixtures with different ratios (Figure 6b). The presence of high amounts of nonpolar amino acids in hordein's and glutelin's primary structures (such as proline, alanine, valine and leucine) induced the aggregation of the proteins at the fish oil droplets surface and allowed the resulting shell to crosslink.

Most of the literature on protein shell microcapsules fabricated through spray drying describes objects with sizes over 1 μm, which presents a barrier for their use in intravenous drug delivery applications. Recent works reported smaller capsules produced by spray drying. To achieve this, a new device (Büchi Nano Spray Dryer B‐90) was proposed to create smaller droplets before the drying step and collect smaller capsules.^[^ ^53^ ^]^ Using this device, Li et al.^[^ ^54^ ^]^ were able to fabricate whey protein and acacia gum shell capsules with a diameter between 350 and 600 nm, respectively, closer to the barrier of 100 nm that defines the transition from microcapsules to nanocapsules. The core of the capsules fabricated by Li's group was composed of vitamin E acetate emulsified in the presence of a nonionic surfactant (Cremophor ELP) before being added to an aqueous solution of the wall material. The mixture was then fed to the spray dryer that generates the powder of capsules. This group also studied how the concentration of the solution to be sprayed affected capsule size and concluded that more diluted samples resulted in smaller capsules. Lower sample concentration (0.1 wt%) resulted in smaller microcapsules with lower size dispersity than higher concentrated samples (1 and 10 wt%).

In brief, the spray drying process allows the fabrication of microcapsules using different proteins with only small adjustments to the protocol. With the right equipment, it can even generate capsules with diameters of less than 1 μm. More information about this specific topic, beyond the protein shell issue, can be found in the review of Arpagaus^[^ ^53^ ^]^ and Nesterenko et al.^[^ ^55^ ^]^

### 2.3 Solid Core and Protein Membrane Microcapsules

Reports on solids being encapsulated by proteins through complex coacervation and spray drying can be found in the literature. However, spray drying publications are neither clear about the structure of the microcapsules, nor about the existence of a solid core encapsulated by a protein membrane or the presence of a matrix structure.^[^ ^56^ ^]^ In addition to these two techniques, layer‐by‐layer (LBL) sequential deposition of oppositely‐charged proteins over a solid microparticle template has been extensively employed as a strategy to fabricate solid core microcapsules. As complex coacervation and spray drying have been already discussed, this part of the review focuses mainly on the LBL technique and just briefly describes the two other techniques.

#### 2.3.1 Complex Coacervation Technique

Following their study of 1964 (see Section 2.2.4), Luzzi and Gerraughty published in 1967 a work showing how initial pH, temperature, ratio of solid to encapsulated materials, quantity of cross‐linker, and final pH affected the complex coacervation of pigskin gelatin and acacia gum into solid core microcapsules. They found that, to be encapsulated, a solid material needs to respect some criteria: 1) it should be insoluble but dispersible in the aqueous medium; 2) it should not exhibit surfactant properties; 3) it should be soluble when exposed to a proper solvent; 4) it should not degrade when exposed to aqueous systems; and 5) the solid particles must be dispersible in either gelatin or acacia solutions. As pentobarbituric acid meets all the mentioned criteria and is easily quantified by spectrometry techniques, it was chosen to be encapsulated.^[43b]^ To prepare microcapsules, Luzzy and Gerraughty used the same protocol as disclosed previously (Figure 4a), substituting the oil droplets by the solid particles.^[43]^

#### 2.3.2 LBL Technique

The LBL technique consists in sequentially depositing macromolecules onto the surface of a template (which can be solid, liquid, or gaseous), one layer at a time. This technique was developed in the 1990s by the Decher's group focusing on electrostatic interactions between macromolecules to assure their deposition onto 2D substrates in an ordered manner.^[^ ^57^ ^]^

During LBL, a first layer of charged macromolecules is built through establishment of favorable ionic interactions with the oppositely‐charged substrate followed by the deposition of a second layer of macromolecules with inverse charges (with respect to the first layer). This process can be indefinitely repeated to generate multiple layers and materials with tunable thickness. Since this method only involves adsorption from a solution, theoretically there are no restrictions over the size or shape (2D or 3D) of the substrate.

**Figure 7:**
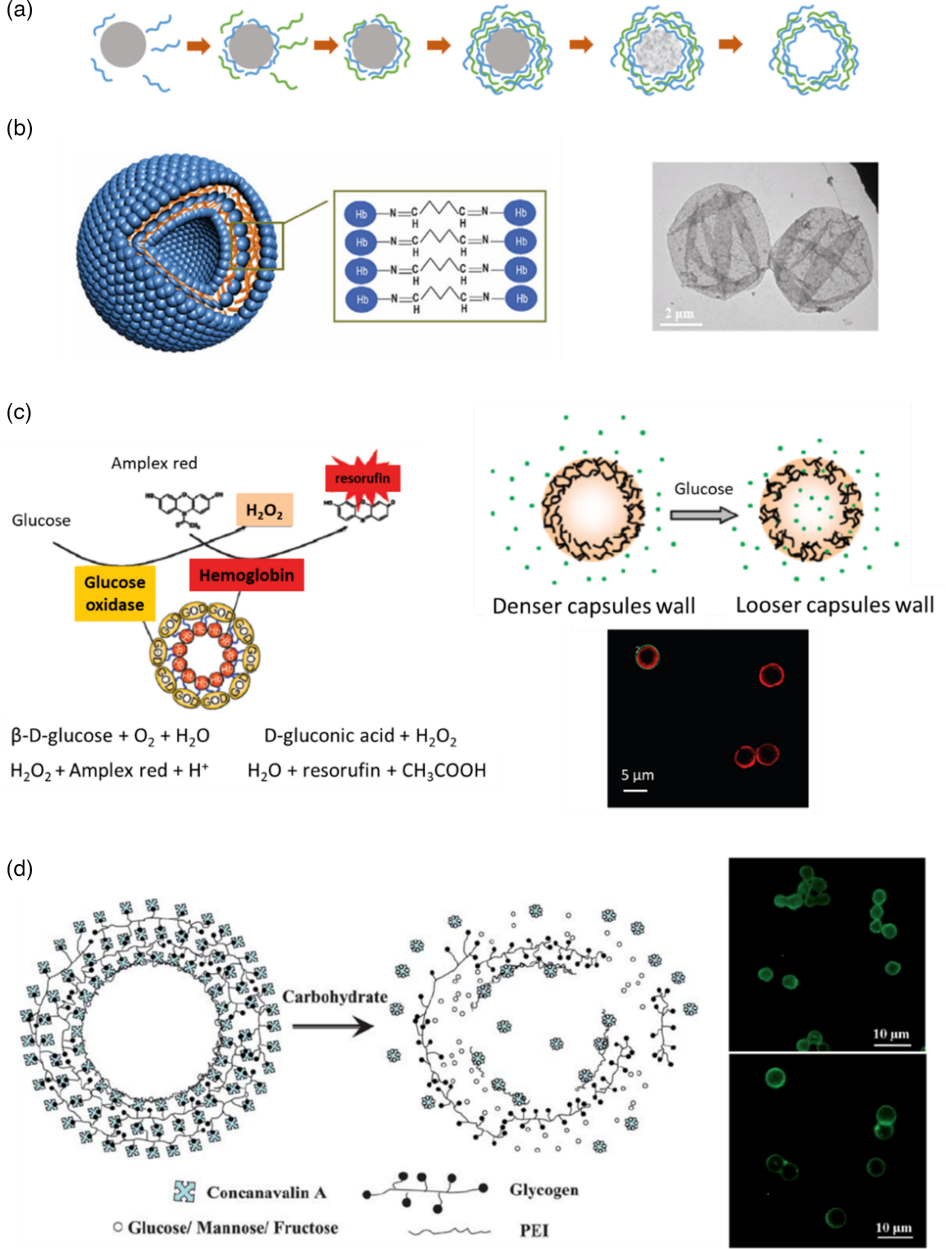
a) Steps of LBL deposition of two different macromolecules on a removable template. Adapted with permission.^[^ ^59^ ^]^ Copyright 2012, The Royal Society of Chemistry. b) Schematic representation of Hb/GA microcapsules fabricated by covalent‐LBL deposition and transmission electron microscopy image of the resulting microcapsules. Reproduced with permission.^[^ ^60^ ^]^ Copyright 2006, Elsevier Inc. c) Schematic of the Hb/GOx capsular structure and of the mechanism of β‐d‐glucose detection by the production of fluorescent resorufin from Amplex red (capsule membrane becomes more permeable in the presence of glucose) together with confocal laser scanning microscopy image of the microcapsules. Reproduced with permission.^[^ ^62^ ^]^ Copyright 2009, American Chemical Society. d) Structure of concanavalin A/glycogen smart microcapsules and their response to carbohydrates together with confocal laser scanning microscopy images of the microcapsules bearing two layers (upper) and seven layers (bottom). Reproduced with permission.^[^ ^64^ ^]^ Copyright 2011, Royal Society of Chemistry.

Reports on the LBL technique being used to decorate the surface of already formed microcapsules with proteins, or to encapsulate cells with protein/polysaccharide hybrid membranes, can be found elsewhere.^[^ ^58^ ^]^ Here, we focus on solid particles being used as template for the deposition of a protein membrane or a protein/polymer hybrid membrane. Different solid particles can be used as a core, but the majority of publications report carbonate (manganese carbonate, MnCO_3_ and calcium carbonate, CaCO_3_) and silica‐based microparticles as templates for the deposition of the membrane. These templates can indeed be easily degraded in the final step of fabrication to produce the final hollow microcapsules. **Figure** **7**a summarizes the different steps of fabrication of hollow microcapsules using the LBL strategy. This technique allows a precise control over the size, shape, and wall thickness of the microcapsules because these parameters depend directly on the original size of the template and the numbers of layers.^[^ ^59^ ^]^

##### Covalent Construction of Multilayered Microcapsules

Microcapsules with covalently cross‐linked membranes tend to be more robust and stable and do not readily disassemble with changes in pH or ionic strength. Several scientific works can be found in the literature on Hb shell microcapsules assembled by the covalent LBL technique. Hb is a protein composed of four polypeptide units (α1, β1, α2, and β2). Each of the subunits presents a globular structure and contains a porphyrin ring called heme that surrounds a Fe^2+^ ion capable of reversibly binding to oxygen. Theses microcapsules have been tested not only as drug carriers but also as blood substitutes, thanks to their unique properties of reestablishing oxygen homeostasis in tissues.

In 2007, Duan et al.^[^ ^60^ ^]^ published their work on Hb protein hollow shells where they used GA to covalently bind several layers of Hb around MnCO_3_ microparticles. The process consisted in coating the MnCO_3_ microparticles (5 μm in diameter) with a layer of poly(ethylenimine) (PEI) so that GA could attach on the surface of the template through the reaction between aldehyde and amine groups. Thanks to the presence of residual aldehyde functions, a layer of Hb could then be deposited and covalently linked on the template. The process was repeated several times to obtain multiple layers of Hb. Finally, the carbonated core was destroyed by adding the chelating agent ethylene diamine tetraacetic acid disodium salt (EDTA) to generate the hollow structures. Figure 7b shows a scheme of the final structure of the microcapsules and the way Hb are bonded together. The mean diameter was not determined by Duan's team, but TEM images showed microcapsules with dimensions around 4 μm. Cyclic voltammetry measurements showed that Hb retained its electroactivity after being assembled into microcapsules.

Two years later, the same group published a very similar protocol to synthesize glucose oxidase (GOx) shell microcapsules cross‐linked with GA.^[^ ^61^ ^]^ GOx is an enzyme produced by fungi and insects that catalyzes the oxidation of glucose. The aim of this work was to replicate the ATP synthase from ADP and inorganic phosphate that takes place in cells. This is a unique process that differs from that of any other enzyme. It entails the transport of protons from one side of the cell membrane to the other. ATP is of extreme importance because it supports nearly all the cellular activities that require some energy. The complex biomimetic structure produced by Duan et al. that replicates the ATP synthase was composed of several layers of GOx, generated by the LBL covalent technique, around a sacrificial MnCO_3_ core. The resulting microcapsules had a diameter around 6 μm. Amperometric measurements confirmed that GOx retained partially its electroactivity after cross‐linking. These structures were coated with a lipid layer containing CF_0_F_1_‐ATPase (the protein responsible for ATP synthesis). When exposed to glucose, protons were generated through hydrolysis and oxidation of glucose by GOx (the gradient of protons between the interior and exterior of the microcapsules being the driving force for ATP synthesis).

The same year, Qi et al.^[^ ^62^ ^]^ reported the preparation of hollow microcapsules with multilayers of Hb/GOx. The goal was to generate stimuli‐sensitive microcapsules to be used as delivery systems or glucose sensors. GOx catalyzes the hydrolysis of β‐d‐glucose into gluconic acid and hydrogen peroxide (H_2_O_2_) and Hb can catalyze the reduction of H_2_O_2_. This hybrid system is sensitive to β‐d‐glucose and its responsiveness can be monitored by the addition of the nonfluorescent dye Amplex red that oxidizes in the presence of H_2_O_2_ into the fluorescent resorufin (see Figure 7c). Five layers of Hb/GA were first deposited onto a MnCO_3_ core (posteriorly removed) initially coated with a first layer of PEI, followed by the construction of five additional layers of GOx (coupled with GA). The fabricated objects had an average diameter around 6 μm. In addition to being able to transform Amplex red into resorufin, the microcapsules membrane permeability could be tuned by adding β‐d‐glucose. The authors proposed that the gluconic acid by‐product locally decreased the pH around the microcapsules, loosening their membranes and enhancing the encapsulation of active substances by diffusion.

Duan et al. published two more articles, in 2012 and 2015,^[^ ^63^ ^]^ exploring oxygen carrying and releasing properties of Hb shell hollow microcapsules generated by the LBL covalent technique. The first study showed that Hb microcapsules functionalized with polyethylene glycol (PEG) retained Hb's oxygen carrying functions. Moreover, GA cross‐linking prevented Hb rapid breakdown from tetramers to dimers, thus increasing capsule half–life and eliminating their nephrotoxicity. In the second study, Hb microcapsules were used as blood replacement to study the effect of radioactive uranyl ion (UO_2_^2+^) on the oxygen‐transporting capability of red blood cells. The high UO_2_^2+^ adsorption capability of Hb microcapsules showed that the ion impairs the oxygen carrying properties and that these microcapsules could be potentially used to remove metal toxin from radiation‐contaminated bodies or from nuclear waste.

##### Noncovalent Construction of Multilayered Microcapsules

The noncovalent interactions used to fabricate and stabilize protein shell microcapsules can be divided into three categories: hydrogen bonding, hydrophobic, and/or electrostatic interactions. Microcapsules prepared by the noncovalent LBL technique are less stable than chemically cross‐linked ones due to the weaker nature of the bonds formed. However, these microcapsules tend to be more sensitive to external stimuli, which make them of special interest to design smart microcapsular systems. In addition, not incorporating cross‐linking agents, such as GA, is highly desirable because they often increase the toxicity of the formulation.^[^ ^64^ ^]^

For example, Zhu et al. published a study in 2011,^[^ ^64^ ^]^ where specific interactions between lectin and carbohydrates were explored to generate microcapsules using the LBL technique. Hydrophobic and hydrogen bonding interactions are weak and reversible, but the numerous binding sites created between the two molecules are capable of stabilizing the microcapsules while offering responsiveness to external stimuli. Zhu's group used concanavalin A (a plant lectin derived from jack beans) and glycogen to generate the capsules’ shells. Their strategy consisted in depositing sequential layers of concanavalin A (first) and glycogen (second) around CaCO_3_ microparticles coated with a first layer of PEI. After removing the core using EDTA, microcapsules were obtained (scheme in Figure 7d). The microcapsules’ size depended on the number of layers, namely, 3.2 μm when two layers were deposited and 5 μm when seven layers were deposited. A number of layers lower than seven resulted in distorted microcapsule shells.

Concanavalin A has strong affinity for glucose, thus the group hypothesized that the free sugars could trigger response from the Concanavalin A/glycogen capsule shells. Zhu's group explored the responsiveness of the capsules to the presence of glucose because it is abundant in human blood. Freshly made capsules could be destroyed within 20 s in the presence of glucose (20 mg mL^−1^).

**Figure 8:**
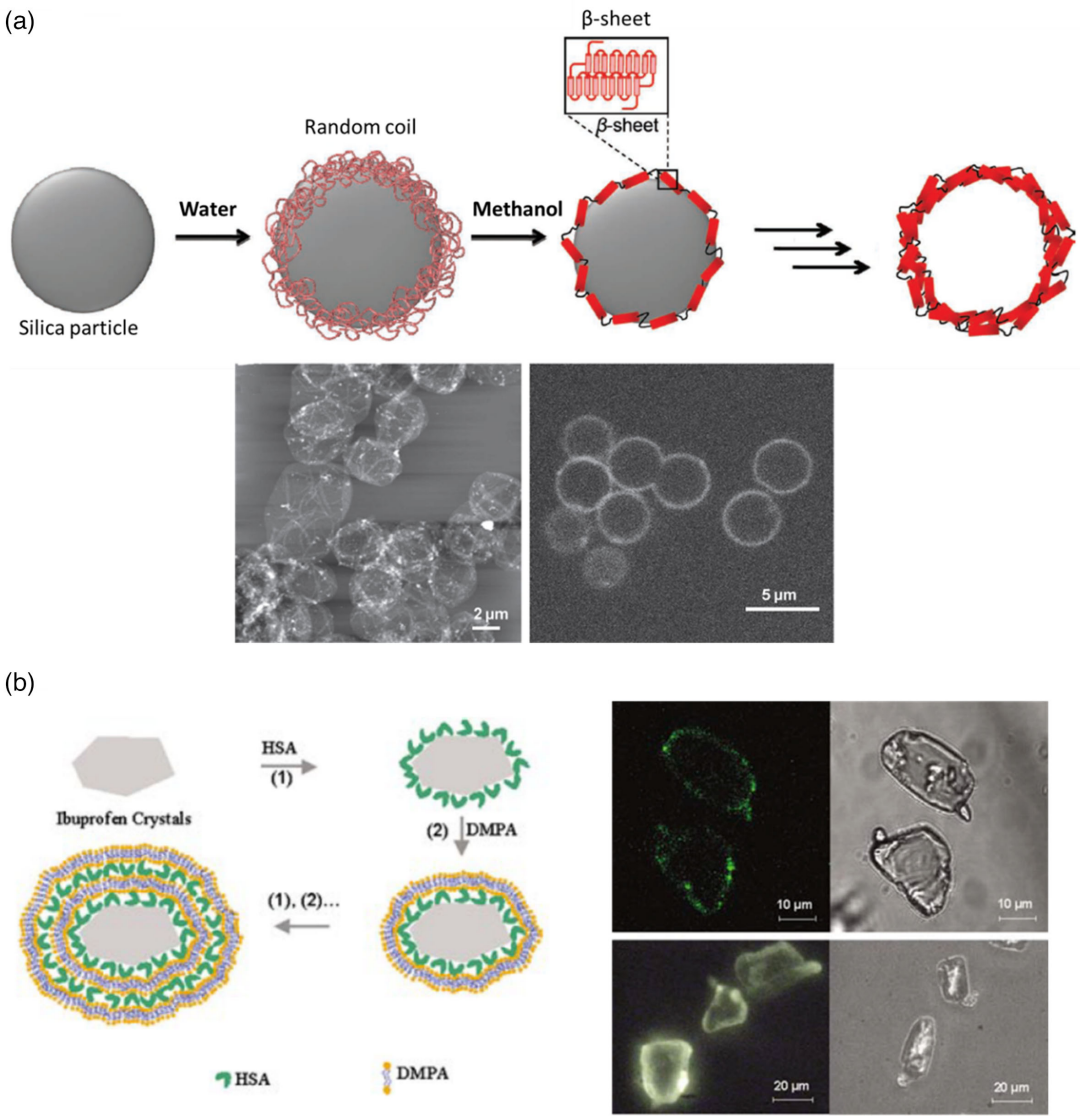
a) LBL silk fibroin microcapsule fabrication (top) and AFM (bottom left) and confocal laser scanning microscopy (bottom right) images of microcapsules bearing five layers. Reproduced with permission.^[^ ^65^ ^]^ Copyright 2011, Wiley‐VCH. b) Different fabrication steps of ibuprofen core and DMPA/HSA shell microcapsules through LBL technique together with optical microscopy images of ibuprofen crystals after adsorption of one layer of HSA (top) and ibuprofen crystals after adsorption of five layers of HSA and DMPA pairs (bottom). Reproduced with permission.^[^ ^67^ ^]^ Copyright 2004, Wiley‐VCH.

In 2011, Shchepelina et al.^[^ ^65^ ^]^ published a work on silk‐on‐silk hollow microcapsules stabilized by physical interactions and hardened by the formation of β‐sheet structures in the proteinaceous membrane. To create the microcapsules, a layer of silk fibroin with a random coil structure was first deposited on silica microparticles in aqueous solution. Then, transfer of these protein‐decorated microparticles in methanol promoted the self‐assembly of the adsorbed proteins into β‐sheets. This procedure was repeated to generate several silk fibroins layers. The silica core was finally removed using hydrofluoric acid (HF) after the desired number of layers was deposited. It is important to note that interactions between the negatively‐charged core and the first positively‐charged protein moieties are mainly electrostatic, while the dominant forces responsible for the deposition of the following layers are mainly of hydrophobic type. The microcapsules produced by Shchepelina's team had a diameter around 3.5 μm and depending of the number of layers, a shell thickness between 10 nm (5 layers) and 54 nm (12 layers) (see microscopy images in **Figure** **8**a for the former). Shell permeability could be tuned to some extent so that thicker shells were less permeable than thinner ones.

One year later, Mertz et al.^[^ ^66^ ^]^ described the fabrication of HSA‐based hollow microcapsules by sequential adsorption of the protein functionalized with isobutyramide derivatives (bromoisobutyramide or isobutyramide) on silica microparticles. In a first step, the silica microparticles were coated with a layer of fluorescently labeled HSA‐rhodamine by protein adsorption from solution. Next, the protein surface was functionalized with one of the isobutyramide derivates before a new HSA coating was deposited again. This protocol was then repeated to obtain as many layers as desired. Noncovalent interactions between HSA and both the amide groups (via hydrogen bonding) and bromine atoms (via halogen bonding, a bond formed between an electrophilic region associated with a halogen atom and a nucleophilic region in a molecule) from the isobutyramide derivative were the driving force for microcapsule generation and stabilization. The authors grew one to five layers of this prefunctionalized HSA over the silica microparticles followed by the removal of the core using HF. Capsule size depended on the diameter of the silica microparticles (either 3 or 5 μm) and the shell thickness was tuned from 6 nm (one layer) to 14 nm (five layers). The authors aimed to use the capsules for drug delivery applications, so cytotoxicity tests were performed on HeLa cells, with no significant cytotoxicity being observed. Microcapsule shell thickness played a role in cellular uptake, with thinner walls being internalized more efficiently by the cells.

In 2004, An et al.^[^ ^67^ ^]^ reported a protocol to synthesize microcapsules where ibuprofen crystals were used as templates to deposit phospholipid/HSA layers. This work is one of the few examples where an active substance was directly used as a core. The phospholipid selected to generate the capsule membrane together with HSA was L–α–dimyristoylphosphatidic acid (DMPA) which can be found in cell membranes. The goal of using these two shell materials was to facilitate the incorporation of membrane‐specific components, such as receptor channels, into the capsule shells for the purpose of molecular recognition. Meanwhile, ibuprofen is an acidic, nonsteroidal, anti‐inflammatory drug that presents low solubility in aqueous solutions at pH lower than 7 but is readily soluble in aqueous solution above this threshold value. This control over the solubility of ibuprofen allows the preparation of microcapsules in acidic solutions and the possibility to release the drug in aqueous solutions at pH 7.4. The microcapsules were fabricated at pH 3.8 where the surface of ibuprofen crystals is negatively charged, the HSA is positively charged (its IEP is 4.8), and DMPA is negatively charged. Considering this charge distribution, HSA can be used as the first layer followed by the sequential deposition of DMPA and HSA layers. As the ibuprofen crystals are not spherical, the resulting microcapsules did not present a spherical structure, as can be seen in Figure 8b. The authors studied the ibuprofen release in a simulated intestine fluid at pH 7.4, verifying that both thicker capsule walls and larger drug crystals decreased the rate of drug release.

The same group published two additional articles in 2005 and 2006^[^ ^68^ ^]^ reporting the fabrication of hollow microcapsules with the same shell composition but using melamine formaldehyde (MF) and MnCO_3_ templates. They further studied the permeability of this new type of membrane at different pHs. As both MF and MnCO_3_ microparticles are positively charged at pH 3.8, An's group changed the order of addition to build the shell layers, starting with a layer of positively charged DMPA followed by the negatively charged HSA. The core was chemically degraded by addition of HF, which the authors hypothesized was responsible for the rough morphology of the resulting microcapsules. This could be the cause of the high permeability presented by the DMPA/HSA membrane for molecules having molecular weights below 40 kDa. When shells containing four DMPA/HSA layers were exposed to a pH of 7.4, permeability was significantly reduced compared to when they were exposed to lower pHs. The authors claimed that these microcapsules show great potential to be used as smart vehicles for drug delivery applications.

Ye et al.^[^ ^69^ ^]^ opted for a different strategy when choosing the proteins used for building the shell of their hollow microcapsules. This group decided to modify silk fibroin (SF) with poly(lysine) (SF‐PL) and poly(glutamic) acid (SF‐PG) to obtain two polyelectrolytes later engaged in the LBL protocol. The modification was induced by diazonium activation of the tyrosine side groups in the SF chains, followed by chemical linkage with polylysine or polyglutamic acid as reported before.^[^ ^70^ ^]^ PEI‐coated silica microparticles were used as template and were destroyed using a solution of HF/NH_4_F after the sequential deposition of SF‐PL and SF‐PG layers. To increase microcapsule stability, 1–ethyl–3–[3–dimethylaminopropyl] carbodiimide hydrochloride (EDC) was used to cross‐link the silk fibroin's functionalized pendent groups before core removal. EDC is typically used to activate COOH in amidification reactions, here mostly between polylysine and poly‐(glutamic acid). The extremely robust silk‐shelled microcapsules were stable over a wide range of pH (from 1 to 12). The permeability of the membrane was pH dependent. At pH 5.5 FITC‐dextran of 2000 kDa could not penetrate the capsules’ membrane. When the pH was shifted to 11.5, the fluorescence was observed in the capsules’ core, indicating that the FITC‐dextran diffused through the membrane. The number of layers also affected shell permeability. Three SF‐PL/SF‐PG bilayer membranes were permeable to FITC‐dextrans of up to 2000 kDa, while nine bilayer membranes only showed permeability to 4 kDa or less.

Following this publication, the same group published another study in 2012,^[^ ^71^ ^]^ where the morphology and micromechanical properties of the microcapsules described above were thoroughly analyzed at different pHs. Significant changes in the physical properties of the membranes were observed under extreme pH conditions (less than 3 and above 11). The group hypothesized that the protonation of carboxyl‐terminal groups on SF–PG (below its pK_a,1_) and the deprotonation of amino groups on the SF‐PL (above its pK_a,2_) reduced the confinement of the silk backbones. This resulted in excessive swelling of the shells and loss of the secondary structure of silk material. Even though microcapsules were not destroyed in these extreme conditions, an increase of shell permeability and a great loss of microcapsule stiffness were observed.

## 3 Routes to Protein Shell Nanocapsules

Thanks to their small size compared to microcapsules, nanocapsules show singular capabilities of bypassing biological barriers while retaining the function of protecting the sensitive cargo. For this reason, their use as drug nanocarriers shall augment the site‐specific dose retention and the bioavailability of drugs. However, to be effective, nanocapsules have to meet certain requirements, such as low toxicity, high loading efficiency, and a sustained release of the cargos.^[^ ^72^ ^]^

Even though proteins are very good candidates for drug delivery systems, the small size of nanocapsules and the complexity of this class of materials result in the need of complex protocols to generate protein‐shelled nanocapsules. The few publications about this type of structures and the fact that most studies date from the two last decades are good indicators of how challenging their synthesis is. Two distinguishable types of protein–based capsular nanostructures can be found in the literature. The first category gathers spherical nanocapsules, stemming from deposition of protein chains onto a template, whereas the second one is based on protein self‐assembled structures that exhibit spherical or nonspherical morphologies. These supramolecular proteinaceous structures coined as “protein cages” have a great variety of shapes and compositions, such as protein viral capsids, bacterial microcompartments, or iron storage protein ferritins.^[^ ^73^ ^]^ These two different types of capsular structures are discussed separately in the following paragraphs.

### 3.1 Protein Shell Nanocapsules by Template‐Driven Fabrication

Most studies used oil emulsions or solid nanoparticles as the nanocapsule core and explored different strategies to generate the membrane. Some of the techniques described to prepare microcapsules are suitable with smaller templates. Other synthetic routes have been specifically developed to take advantage of proteins intrinsic functions and design enhanced delivery systems.

#### 3.1.1 LBL Technique Involving Sacrificial Solid Templates

In 2011, Yang et al.^[^ ^74^ ^]^ used the covalent LBL technique to coat mesoporous silica nanoparticles with Hb/GOx shells. This study followed the extensive work the group did on Hb/GOx shell microcapsules summarized in Section 2.3.2. The deposition technique was performed in the same way, with GA to cross‐link the layers, the biggest change being the mesoporous silica nanoparticles used as core. These nanoparticles are particularly significant in medical and biological fields where they are used as cell markers, gene transfection reagents, or MRI contrast agents. The nanocapsules synthesized by this method exhibited a mean diameter between 100 and 150 nm. The shell proteins kept their enzymatic activity, which resulted in controlled fluorescent activity sensitive to the presence of glucose and showed good internalization by cells. All these features make the Hb/GOx nanocapsules good candidates to be used as cell markers.

#### 3.1.2 Deposition and Cross‐Linking of Proteins onto Sacrificial Solid Templates

**Figure 9:**
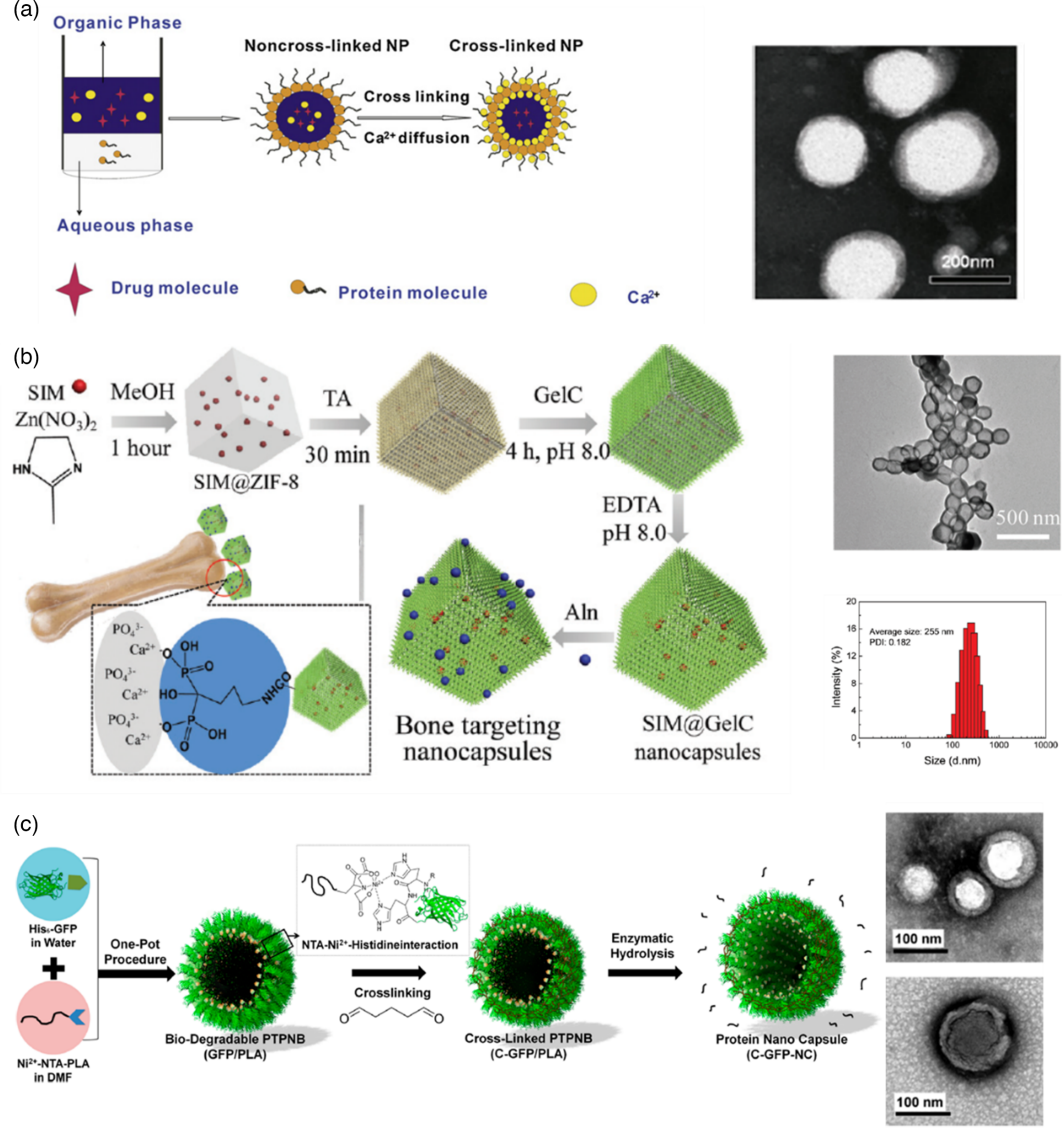
a) Process used to prepare SPI, WPI, and β‐lg cross‐linked nanocapsules and transmission electron microscopy images of SPI nanocapsules. Reproduced with permission.^[^ ^78^ ^]^ Copyright 2013, Elsevier B.V. b) Route to drug‐loaded bone targeting nanocapsules (left), TEM image (top right), and DLS size distribution (bottom right) of the obtained nanocapsules after 14 days of storage. Reproduced with permission.^[^ ^75^ ^]^ Copyright 2017, Royal Society of Chemistry. c) Preparation steps of hollow nanocapsules with GFP shell (left) and TEM images of cross‐linked GFP/PLA nanocapsules before (top right) and after (bottom right) core removal. Reproduced with permission.^[76a]^ Copyright 2018, American Chemical Society.

In 2017, Wang et al.^[^ ^75^ ^]^ reported the fabrication of simvastatin (SIM)–loaded nanocapsules with catechol‐modified gelatin (GelC) as the wall material, functionalized with amine‐containing alendronate (Aln) ligand that specifically binds to hydroxyapatite. These colloids are intended to be used as carriers for bone‐targeted drug delivery because simvastatin is a lipophilic drug used for the treatment of osteoporosis. The metal‐organic framework ZIF–8 (possessing a polyhedral shape) was used as sacrificial template where SIM was encapsulated via coprecipitation in methanol (SIM@ZIF–8). The SIM@ZIF–8 particles were then coated with tannic acid (TA), through coordination interaction with the zinc ions present on the particles’ surface, which improved hydrophilicity and allowed the dispersion of SIM@ZIF–8 in the aqueous solution of GelC. The deposition of the GelC onto the TA‐modified SIM@ZIF‐8 nanoparticles was driven by hydrogen bonding, hydrophobic interactions, and *π*–*π* stacking between the galloyl group from the TA and catechol from the GelC. The ZIF‐8 core was then removed by exposure of the objects to EDTA, leaving the SIM inside the shell. Finally, Aln was covalently attached on the surface of nanocapsules via Michael addition and Schiff base reactions between the amine and catechol groups (see **Figure** **9**b). The capsules had a mean diameter of 238 nm (255 nm after 14 days storage) and presented a drug encapsulation efficiency of 77%. In addition, sustained release of the drug was verified. The objects could be effectively internalized by osteoblasts with minimal toxicity to myoblasts at concentrations below 100 μg mL^−1^. High hydroxyapatite binding affinity and bone accumulation were observed in vitro and in vivo for the Aln functionalized GelC nanocapsules.

In 2018, Lee et al.^[^ ^76^ ^]^ published two studies on histidine‐tagged green fluorescence protein (His_6_–GFP) shelled nanocapsules where the protein was immobilized with targeted orientation on a polymeric template. The authors claimed that controlling protein orientation preserved its function. To achieve this type of oriented structure, the group synthesized nickel(II) nitrilotriacetic acid end–functionalized poly(lactic acid) (Ni^2+^–NTA–PLA) and formulated it into spherical nanoparticles as templates. Specific interactions between the NTA‐Ni^2+^‐His polymer chain ends and multihistidine tags on proteins promoted the generation of the GFP shell around the PLA nanoparticles. After cross‐linking the GFP chains using GA, the PLA core was removed by enzymatic degradation. Figure 9c illustrates the process. Lee's group tuned the dimensions of the nanocapsules by controlling the templates’ size, which allowed them varying the mean diameters from 62 to 184 nm. The nanocapsules permeability was evaluated by encapsulating rhodamine B isothiocyanate–dextran (RITC‐dex) of distinct molecular weights (10 and 70 kDa). The RITC‐dex with 10 kDa penetrated deeply into the nanocapsules while the 70 kDa version could not efficiency penetrate the nanocapsules shells, indicating a shell pore cutoff inferior to 70 kDa. The fluorescent dye sulforhodamine 101 was encapsulated with an encapsulating efficiency of 5% and loading capacity of 22%.

#### 3.1.3 Complex Coacervation Emulsification Technique

Complex coacervation, discussed in Section 2.3.1, can also be used to fabricate protein shell nanocapsules from natural proteins. In 2014, Lv et al.^[^ ^77^ ^]^ fabricated heat‐resistant nanocapsules with a gelatin/acacia gum shell and a jasmine oil core. The final goal was to encapsulate flavors to protect them even at high temperatures. Nanocapsules with a diameter around 70 nm were synthesized by emulsifying the oil using a high‐speed dispersing machine (capable of generating nm scale oil droplets) followed by the addition of the shell materials and subsequent acidification of the solution (to pH 4.8) to trigger the coacervation. The cross‐linking was performed using transglutaminase in alkaline conditions, which is capable of catalysing acetyle transfer reactions that create covalent linkages between proteins. The most frequent linkage happens between the ϵ‐amino group present in lysine residues and the γ‐carboxamide group present in glutamine residues. These nanocapsules presented high stability at room temperature as well as in humid environments at 80 °C, suggesting that they could be of interest as delivery vehicles for flavors.

#### 3.1.4 Miniemulsion Technique

In 2013, He et al.^[^ ^78^ ^]^ reported the fabrication of SPI, whey protein isolate (WPI), and β‐lactoglobulin (β–lg) shell and oily core nanocapsules, physically cross‐linked by ionic pairing between Ca^2+^ ions (present at the interface of the oily core and the aqueous phase) and the carboxyl groups of the proteins. Fenofibrate, a pharmaceutical drug used to treat abnormal blood lipid levels, was encapsulated in the core. To fabricate the nanocapsules, the oil Labrafil M 1944CS containing the drug and 4 m of CaCl_2_ (dispersed in the oil phase by vortex mixing) was added to an aqueous solution containing the protein and the mixture was homogenized using an Ultra‐Turrax blender. The formation of the miniemulsion and cross‐linking of the shell happened simultaneously without addition of a coacervation/precipitation agent. The CaCl_2_ salt present in the core dissociated into Ca^2+^ and Cl^−^ ions, which resulted in the formation of protein‐Ca‐protein bridges at the o/w interface, packing the protein chains around the core and leading to further protein cross‐linking by interaction between carboxyl and amino groups (protocol illustrated in Figure 9a). He's group fabricated nanocapsules as small as 100 nm using this strategy with a 50% drug loading capacity and 90% loading efficiency.

#### 3.1.5 Inverse Miniemulsion Technique

**Figure 10:**
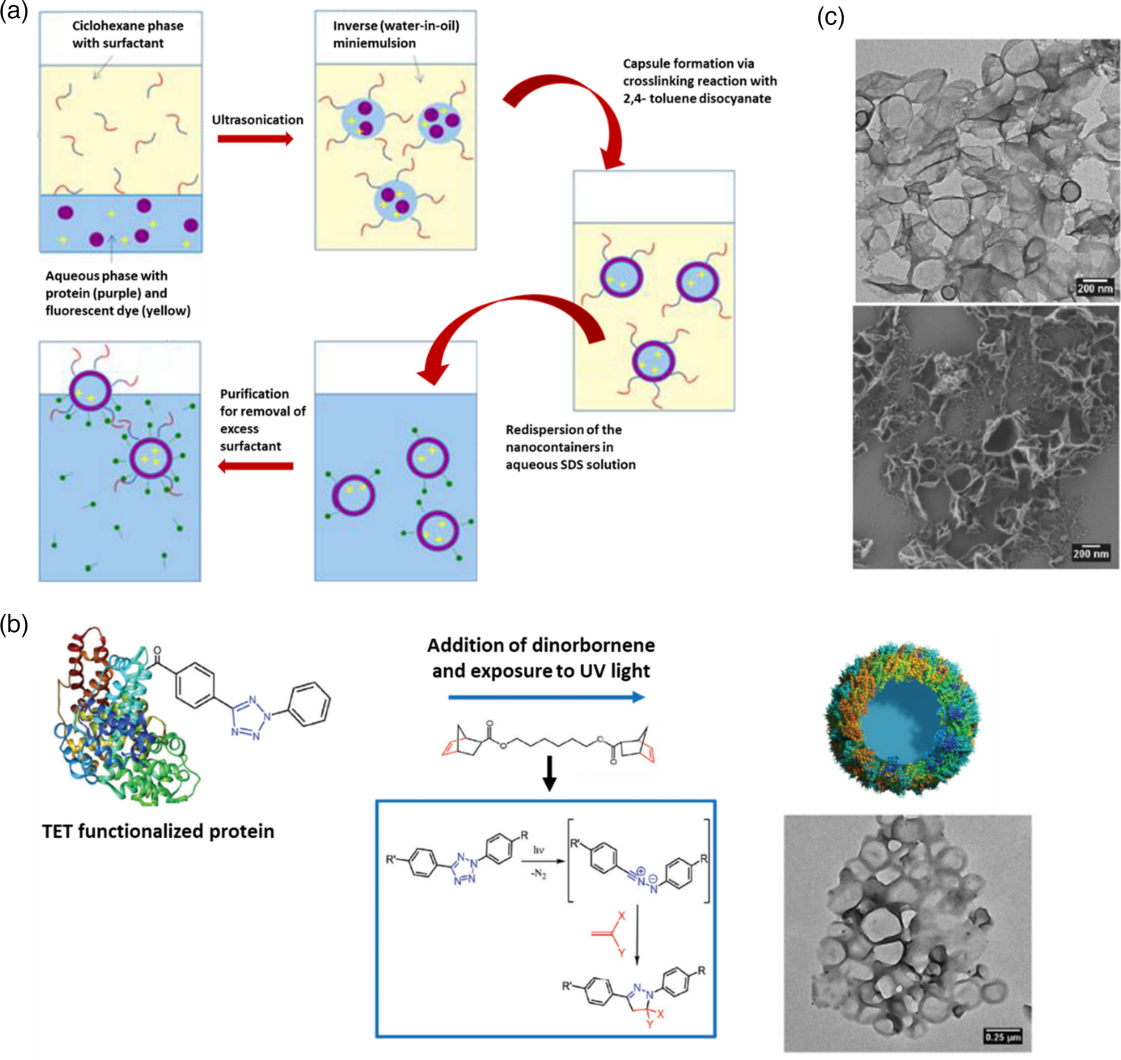
a) Synthesis of protein nanocapsules via cross‐linking reaction in an inverse miniemulsion. Reproduced with permission.^[^ ^72^ ^]^ Copyright 2015, American Chemical Society. b) Reaction mechanism involved in the preparation of protein nanocarriers by cross‐linking of protein‐TET conjugates with dinorbornene in an inverse emulsion and TEM image of the obtained nanocapsules. Reproduced under the terms of the CC‐BY 3.0 license.^[79b]^ Copyright 2017, The Authors, published by The Royal Society of Chemistry. c) TEM (top) and SEM (bottom) images of NS5A nanocapsules. Reproduced with permission.^[79a]^ Copyright 2016, Elsevier Ltd.

Landfester's group^[^ 72, 79 ^]^ developed a protocol that takes advantage of a chemical reaction at the interface of water droplets, generated as an inverse emulsion, to create nanocapsules with a water core and a protein shell. Hydrophilic compounds can be encapsulated with high efficiency within this aqueous core. So far, all the protocols that allowed the formation of water‐filled capsules relied on the use of solid nanoparticles coated and posteriorly destroyed, often using dangerous and toxic chemicals. The inverse miniemulsion was first generated by ultrasound in the presence of a polymeric surfactant, poly–(ethylene–*co*–butylene)–*b*–(ethylene oxide) (P((E/B)‐*b*‐EO) and the protein that ultimately forms the nanocapsule shell (see **Figure** **10**a).

In 2015, Landfester and collaborators^[^ ^72^ ^]^ reported nanocapsules with a BSA or ovalbumin (OVA) shell generated by a polyaddition reaction between the cross‐linker 2,4‐ toluene diisocyanate (TDI) and the nucleophilic groups of the proteins (hydroxyl and amine groups) at the interface of water nanodroplets. The resulting OVA‐ and BSA‐based nanocapsules had a mean diameter between 160 and 190 nm and presented loading efficiency (tested with the hydrophilic dye SR101) of 97% and 88%, respectively. The stability in blood plasma together with the degradability when treated with serine protease trypsin suggests that these nanocapsules can be used as antigen carriers for vaccine development.

Two years after, the same group published a study on the construction of albumin‐shelled nanocapsules through inverse miniemulsion using photoclick tetrazole‐ene cycloaddition for shell stabilization.^[79b]^ Triggered by light, this reaction allowed the preparation of the nanocarriers under mild conditions while preventing the loss of bioavailability of the antitumor and antiviral encapsulated active (Resiquimod R848). To perform the cross‐linking reaction, 4–(2–phenyl–2 H‐tetrazol–5–yl) benzoic acid (TET) was first attached to OVA and HSA by Steglitch amidation. The modified proteins dissolved in an aqueous solution were mixed with hexadecane containing the P((E/B)‐*b*‐EO) surfactant and the two phases were homogenized using an ultrasound probe. Addition of dinorbornene cross‐linker to the miniemulsion and subsequent exposure to UV triggered the reaction between the TET moieties and the cross‐linker at the oil/water interface, generating nanocapsules with a mean diameter of 300 nm and a zeta potential of ‐30 mV (reaction mechanism illustrated in Figure 10b). The nanocapsules presented low toxicity, high drug encapsulation ratio (90%), good stability in blood plasma, and were able to store the drug for several months without unwanted release. In addition, exposing the nanocapsules to serine protease trypsin resulted in fast degradation of the protein corona and release of the cargo.

In 2016, the group of Landfester^[79a]^ reported water‐filled hepatitis C virus nonstructural protein 5 A (NS5A)‐based nanocapsules having monophosphoryl lipid A (MPLA) adsorbed on their membrane. The goal of producing this kind of structures was to test their in vitro and in vivo interactions with liver dendritic cells and their potential to induce intrahepatic immune responses to check their potential as antigen carriers in vaccines for liver diseases. Using the specific antigen directly as nanocapsule shell can prevent common side effects such as the generation of immunity against the carriers. Recombinant technology was first used to express the NS5A protein using yeast *Pichia pastoris* strain X‐33 cells with the pPICZα A vector. An inverse emulsion, containing the P((E/B)‐*b*‐EO) surfactant, was then used as a template to generate the protein shell stabilized through cross‐linking with TDI. Adsorption of MPLA was performed after purification of the sample by mixing the aqueous dispersion of nanocapsules together with the MPLA dissolved in DMSO. Dynamic light scattering (DLS) of the nanocapsules dispersed in water showed an average diameter of 428 nm (microscopy images in Figure 10c). The HCV‐specific NS5A antigen nanocapsules functionalized with the adjuvant MPLA proved to be capable of targeting liver–resident antigen‐presenting cells leading to an efficient maturation and activation.^[79a]^

#### 3.1.6 Nanoprecipitation Technique

Nanoprecipitation is a simple “bottom‐up” approach that relies on the supersaturation of a hydrophobic solute (such as an oil, first dissolved in a water‐miscible solvent) upon addition of water. To generate a (meta)stable emulsion that will work as template, phase diagrams are established to identify the domains of interest, e.g., Ouzo and surfactant‐free microemulsion (SFME) regions.^[^ ^80^ ^]^ This information is then used to settle conditions of solvent shifting where the solute is spontaneously emulsified and assembled into submicrometer templates, onto which hydrophilic polymers or protein chains (dissolved in the aqueous phase) precipitate to form a stable shell. Following this route, oil‐filled nanocapsules have been constructed from a range of vinyl‐based polymers and polysaccharides.^[^ ^81^ ^]^ The first report of nanoprecipitation process used to generate nanocapsules from artificial proteins is the work of Ramos et al. in 2021, where a modular fusion protein designed based on squid suckerin proteins was used to form the nanocapsules’ shell.^[^ ^82^ ^]^

Suckerins are a family of structural proteins that form the building blocks of sucker ring teeth^[^ ^83^ ^]^ lining up the arms and tentacles of squids and cuttlefish. They are characterized by a block copolymer architecture reminiscent of silk fibroins, consisting of β‐sheet‐forming domains intervened by longer flexible segments. The β‐sheet domains are enriched in the basic residue His (about 10 mol%), allowing to solubilize the protein under acidic pH by charge–charge repulsion of the positively charged His side‐chains. By screening suckerin‐19 with kosmotrophic salts such as NaCl and KCl, charge screening occurred, resulting in self‐assembly of β‐sheet‐enriched nanoparticles. Depending on the conditions (salt concentration, temperature), the average particle size varied from ≈190 to 450 nm with a low polydispersity index.^[^ ^84^ ^]^ Even though nanoparticles could be readily prepared using suckerin proteins, their moderate solubility at neutral pH was not compatible with the nanoprecipitation technique. For this reason, Ramos et al. decided to design a fusion protein consisting of a central squid suckerin‐derived peptide (offering structural stability) flanked by termini from silk protein domains that enhanced solubility at physiological pH. The improved solubility of this fusion protein allowed the establishment of conditions where a metastable emulsion of miglyol or hexadecane was simultaneously generated, with the protein precipitating preferentially at the surface of the oil droplets. Using this approach nanocapsules with a size around 190–250 nm were built.^[^ ^82^ ^]^

### 3.2 Protein Cages

**Figure 11:**
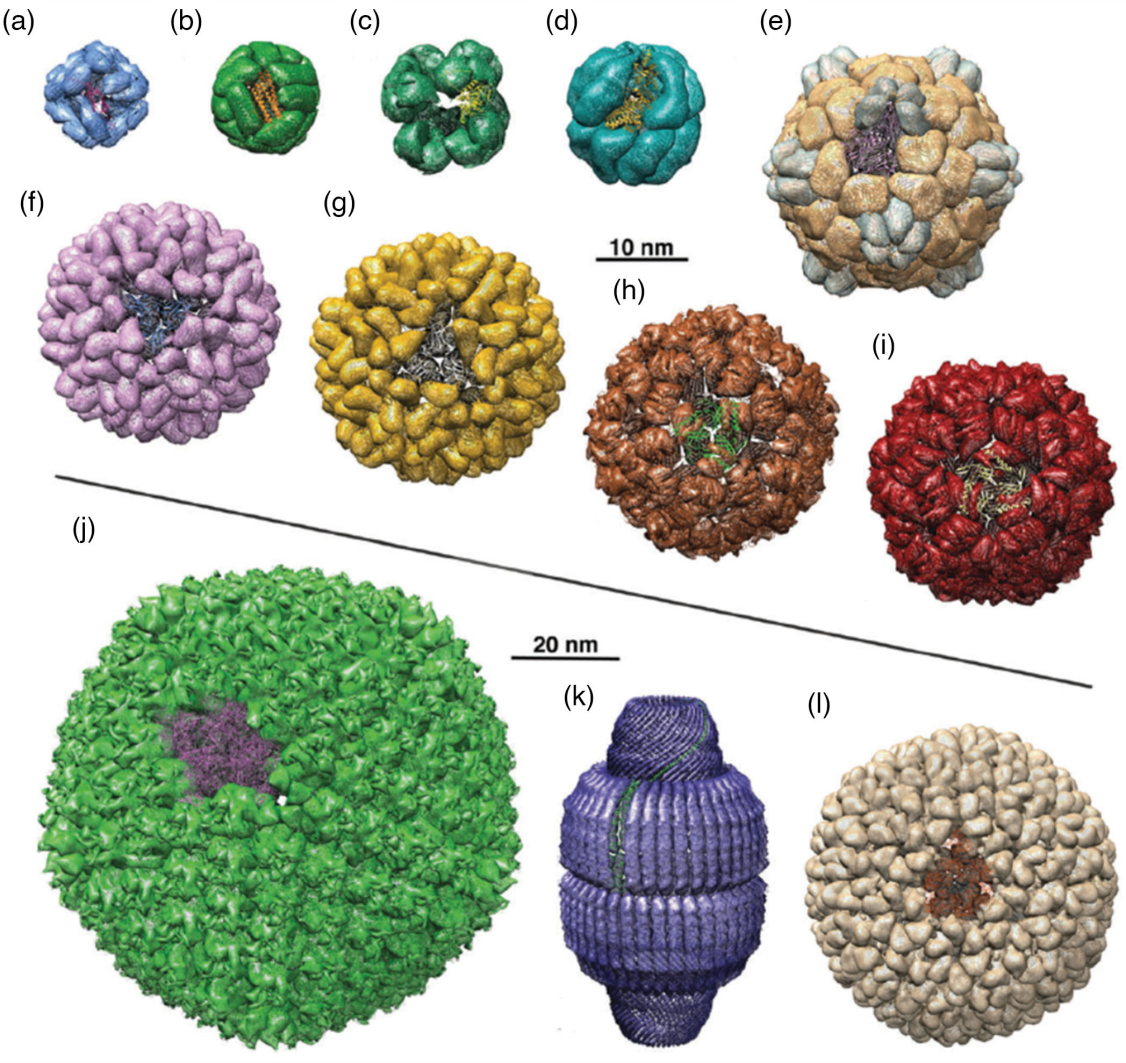
Protein cages ribbon structure: a) small heat‐shock protein; b) apoferritin; c) pyruvate dehydrogenase multienzyme complex; d) thermosome; e) cowpea mosaic virus; f) brome mosaic virus; g) cowpea chlorotic mottle virus; h) Qβ bacteriophage; i) bacteriophage MS; j) human adenovirus; k) vault particle; l) bacteriophage P22. Reproduced with permission.^[^ ^73^ ^]^ Copyright 2016, Royal Society of Chemistry.

Protein cages are hollow proteinaceous structures composed of self‐assembled protein subunits with a nearly monodisperse size distribution. These types of structures can be made of a single protein or multiple proteins and are often fabricated in living hosts, such as *E. coli*, plants, or mammalian cells. Protein cages can be divided in two categories, namely, viral and nonviral protein cages. Examples of nonviral protein cages are ferritins, vaults, heat‐shock proteins, chaperonins, lumazine synthase, encapsulins, and bacterial microcompartments. Examples of viral cages are the tobacco mosaic virus and the M13 bacteriophage (rod‐shaped viruses), cowpea mosaic virus, cowpea chlorotic mottle virus, bacteriophage MS2, and adenovirus (icosahedral structures). **Figure** **11** illustrates the diversity of the protein cages mentioned here.^[^ 73, 85 ^]^

These types of structures offer the possibility of transporting and protecting drugs, metal nanoparticles, macromolecules, catalysts, and many other guests inside carriers with very well‐defined morphologies and sizes. Four different strategies have been explored to encapsulate molecules in or on protein cages. The interior of the proteins cages is accessible via multiple pores of defined sizes (e.g., 0.4 nm for ferritin, 3 nm for heat shock proteins and 10 nm for P22 capsid) through which small molecules can smuggle in by diffusion. As the size of these pores is constant for each type of protein cage, they can be used as size‐exclusion passages that only allow smaller substances than the pore size to be encapsulated. The use of genetic engineering to precisely modify the protein that forms the cage at specific sites allows for chemical conjugation of molecules of interest, both in the interior and in the exterior of the cage. For example, the insertion of cysteine or nonnative amino acids can act as attachment points. Physical interactions, such as hydrophobic and electrostatic interactions between the cage and the molecule to be loaded, have also been explored as an encapsulation mechanism. Finally, some of the protein cages like ferritins possess the property of disassembling and reassembling when submitted to specific conditions (e.g., pH shift) which can be used to encapsulate and release substances of interest.^[^ ^73^ ^]^

Here, only the works where a substance was encapsulated for biomedical applications are discussed. As the protein cages sometimes present immunogenic responses and low blood circulation times, polymer chains like PEG chains are often conjugated together with the protein cage by means of available reactive groups on the proteins or by introducing the desired reactive groups through recombinant technologies.^[^ ^73^ ^]^ The reviews of Molino et al.^[^ ^85^ ^]^ and Rother et al.^[^ ^73^ ^]^ are good reference points for the work done with protein cages in drug and gene delivery until 2016. For this reason, the cases studied on these reviews are summarized in **Table** **2** and a few more recent works are further discussed here.

**Table 2:** Active substances encapsulated in viral and nonviral protein cages and encapsulation strategies

Protein cage	Drug	Encapsulation strategy	References
Nonviral protein cages			
Heat shock protein	Dox	Hydrazone derivative of doxorubicin linked to the interior surface of HspG41C via coupling of maleimide and thiol functionalities.	[122]
Ferritin/Apoferritin	Duanomycin	pH sensitive disassembly and reassembly mechanism used to load the drug.	[123]
	Cisplatin	pH sensitive disassembly and reassembly mechanism used to load the drug.	[124]
	5‐Fluorouracil	Diffusion of the drug through the pores of the cage's shell.	[125]
	Dox	Copper‐Dox complexes capable to bind to apoferritin binding sites.	[126]
E2 subunit pyruvate dehydrogenase	Dox	(6‐Maleinimidocaproyl) hydrazone derivative of Dox covalently coupled to the interior cavity of the cage.	[127]
	CpG	CpG conjugated to inner cysteine residues, forming acid‐labile hydrazone bonds.	[128]
Viral protein cages			
Adenovirus	Bleomycin	Bleomycin A5 hydrochloride crosslinked to the protein cage.	[129]
	Paclitaxel	Drug conjugated to the exterior of folate‐modified adenovirus cages.	[130]
Cowpea mosaic virus	Dox	Dox molecules covalently bound to external surface carboxylates.	[131]
	Proflavin	Interaction of proflavin with the CPMV's encapsulated RNA molecules. Noncovalent infusion of the drug.	[132]
Bacteriophage MS2	siRNA	Encapsulation by formation of covalent bonds.	[133]
	Dox	Encapsulation by formation of covalent bonds.	
	5‐Fluorouracil	Encapsulation by formation of covalent bonds.	
	miRNA	Covalent conjugation between the amino group of MS2 and the cysteine group of the Tat47‐57 peptide (miRNA).	[134]
Qβ bacteriophage	EGF	C‐terminal genetic fusion to the Qβ capsid protein subunits. EGF displayed on the exterior of the capsid.	[135]

#### 3.2.1 Nonviral Protein Cages

##### Small Heat‐Shock Proteins

Heat‐shock proteins protect cells from environmental and physiological stresses. They are therefore produced when the cells are exposed to elevated temperatures. Some of these proteins have cage‐like structures, alike the small heat‐shock protein from *Methanococcus jannaishii* Hsp16.5 (Figure 11a). It is composed of 24 protein subunits that self‐assemble into 12 nm hollow structures with a cavity of 9 and 3 nm‐sized pores. This cage can withstand relatively harsh conditions such as a pH of 11 and temperatures up to 70 °C.^[^ ^73^ ^]^

**Figure 12:**
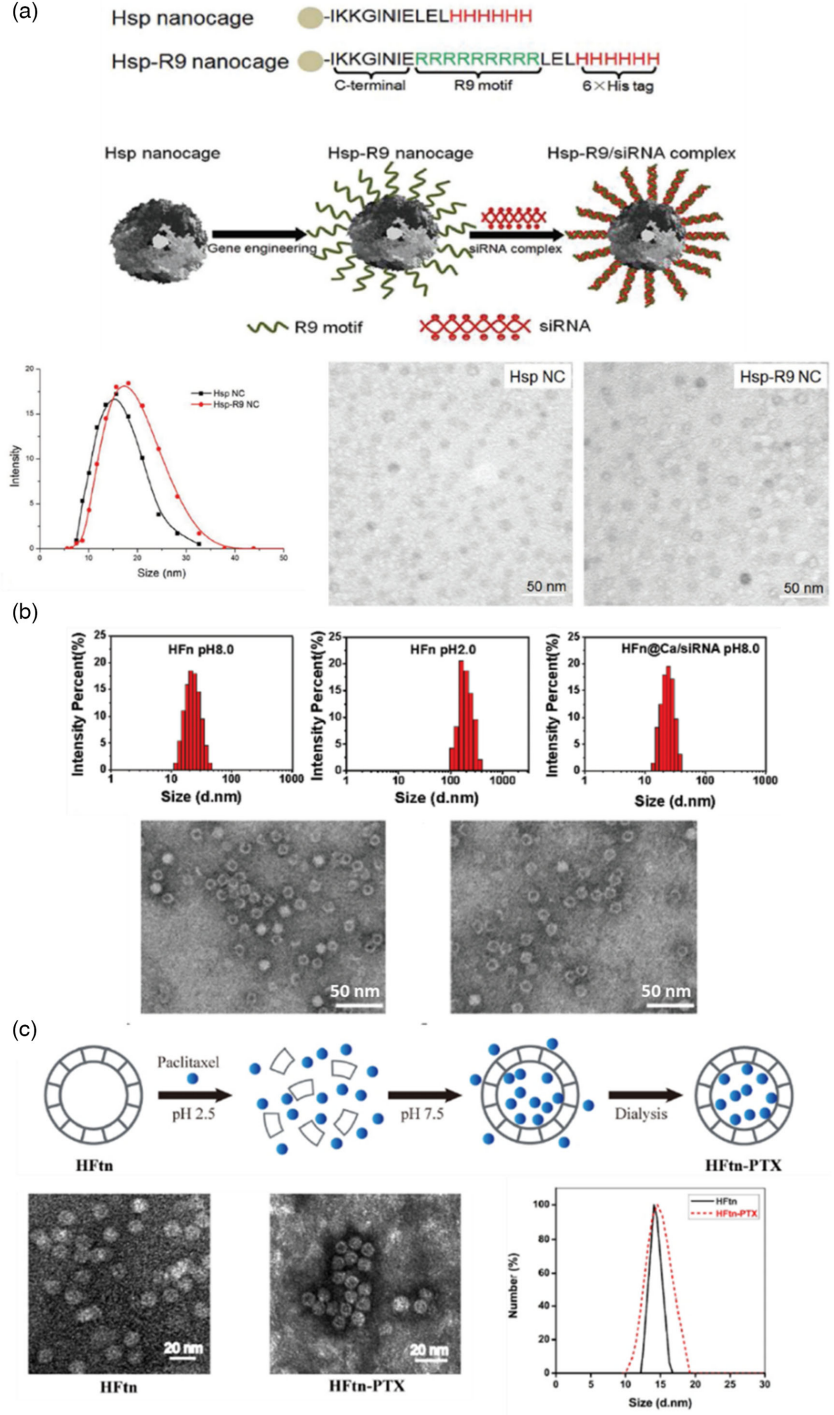
a) Adsorption of polyarginine onto the Hsp16.5 nanocage (top) and DLS (bottom left) and TEM characterizations of Hsp16.5 (bottom middle) and Hsp16.5‐R9 (bottom right). Reproduced with permission.^[^ ^86^ ^]^ Copyright 2018, Wiley‐VCH. b) DLS spectra of empty human apoferritin (HFn) at pH 8 (top left), pH 2 (top middle) and of apoferritin containing siRNA and calcium phosphate (HFn@Ca/siRNA) at pH 8 (top right). TEM image of HFn (bottom left) and HFn@Ca/siRNA (bottom right). Reproduced with permission.^[^ ^88^ ^]^ Copyright 2020, Royal Society of Chemistry. c) Scheme of the encapsulation mechanism of paclitaxel into apoferritin cages (top), TEM images, and DLS spectra (bottom) of empty (HFtn) and filled (HFtn‐PTX) cages. Reproduced with permission.^[^ ^89^ ^]^ Copyright 2019, American Chemical Society.

In 2018, Guan et al.^[^ ^86^ ^]^ used a mutant Hsp16.5 as siRNA delivery system. The hypothesis was that by complexing siRNA with the protein cage, the gene material would be protected from degradation and would deliver its load into cells in better conditions. The group modified Hsp16.5 through recombinant technology by introducing polyarginine peptides at the C‐terminal sequence of the protein cages subunits (Hsp16.5‐R9, **Figure** **12**a) that are exposed to the exterior of the protein cage. The addition of the peptides had minor effect on the cage's final structure, with only a small change in size being observed between the Hsp16.5 (12.1 nm) and the mutant form (14.8 nm). In contrast, the addition of nine polyarginine residues drastically changed the zeta potential from a relative neutral state to +27.2 mV. Taking advantage of the presence of the charged peptides at the surface of the protein cage, negatively‐charged siRNA targeting EGFP were subsequently complexed to the cages through electrostatic interactions. The group tested the efficacy of the siRNA (targeting EGFP) complexed to Hsp16.5‐R9 for gene silencing by transfecting it to eGFP‐expressing HeLa cells. This experiment allowed the determination of the complex binding efficiency by observing the fluorescence variation of the expressed GFP proteins. A reduction in fluorescence efficiency indicated that the siRNA was able to impair the expression of GFP in the cells. A more pronounced reduction in fluorescence was observed when the Hsp16.5‐R9 containing siRNA was administered to the cells compared to free siRNA, confirming that the system is an improvement to the use of free siRNA.

##### Ferritin and Apoferritin

Ferritins play an essential role in regulating iron levels and protecting animals, plants, and bacteria against oxidants. Their natural function is the biomineralization of Fe^2+^ to Fe_2_O_3_·H_2_O. Mammalian ferritins are made of 24 protein subunits that form a spherical cage with a diameter of 12 nm. In their core, native ferritins concentrate iron ions that can be easily removed, resulting in an empty cage called apoferritin (Figure 11b). The two structures are very robust and withstand temperatures of up to 85 °C and pHs ranging from 2 to 9. In addition, ferritins are easily accessible from natural sources and recombinant expression. Their endogeneity and excellent biocompatibility are also of great interest. Finally, the fact that the cage structure can be disassembled at low pH (pH 2) and reassembled at neutral pH is an interesting possible release trigger.^[^ ^87^ ^]^ All together, these features of ferritin and derivatives make them popular nanocarriers for drug delivery.^[^ ^73^ ^]^

Huang et al.^[^ ^88^ ^]^ encapsulated siRNA, especially designed to silence the epidermal growth factor receptor, in protein cages made of human apoferritin H subunits (recombinantly expressed using the vector pET‐30a transformed into *E. coli*). The group encapsulated the siRNA together with mineralized calcium phosphate using the disassembly/reassembly mechanism previously mentioned. The salt CaCl_2_ and the siRNA were added into a solution of apoferritin at pH 2 followed by a pH adjustment at 8, by addition of NaOH, which induced apoferritin cage assembly with concomitant encapsulation of siRNA and Ca^2+^ cations inside the cavity. Posterior addition of Na_2_HPO_4_ nucleated the mineralization of Ca^2+^ and PO_4_^3−^ into calcium phosphate. Figure 12b shows DLS and TEM characterizations of the empty apoferritin and apoferritin containing the salt and SiRNA.^[^ ^88^ ^]^ The encapsulation efficiency of siRNA depended on the siRNA/apoferritin ratio. A ratio of 1:8 achieved the maximum encapsulation efficiency of 76% while 1:4 resulted in 21% and 1:10 in 34%. The group also tried to encapsulate siRNA in the absence of Ca^2+^, which resulted in an encapsulation efficiency of 46% (for the 1:8 ratio), proving that Ca^2+^ plays a strong role in the encapsulation process. Besides improving encapsulation efficiency by neutralizing the negative charges of the siRNA, Ca^2+^ was used to generate mineralized calcium phosphate inside the apoferritin cage, which has been demonstrated to improve the endosomal escape ability of nanocarriers.

Li et al.^[^ ^89^ ^]^ used the same strategy to encapsulate paclitaxel inside the human apoferritin cage (H subunits recombinantly expressed using vector pET‐20b (+) transformed into *E. coli*). As this antitumor drug is insoluble in water, it was dissolved in ethyl alcohol before being added at an apoferritin/paclitaxel ratio of 1:200 into the apoferritin solution at pH 2.5 (to ensure the apoferritin was disassembled). The pH was then adjusted to 7.5 to induce apoferritin self–assembly into a cage and encapsulate paclitaxel (scheme in Figure 12c). Free paclitaxel molecules were removed by dialysis. Encapsulation efficiency and loading capacity were determined to be 20.1 and 18.4%, respectively. These nanocarriers displayed lower cytotoxicity and higher therapeutic efficiency than the free drug.

#### 3.2.2 Viral Protein Cages

##### Adenovirus

The human adenovirus (Figure 11j) is a double‐stranded DNA virus that measures 95 nm from vertex to vertex when self‐assembled into a capsid. It presents an icosahedral symmetry composed by three main proteins: 720 subunits that form 240 hexon trimers, 12 pentagon‐shaped penton‐base pentamers that are all centered on the vertices of the cage, and 12 fiber trimers that are interconnected with the penton–base pentamers. As it is non‐oncogenic, it can be used in gene and cancer therapy.^[^ ^73^ ^]^

**Figure 13:**
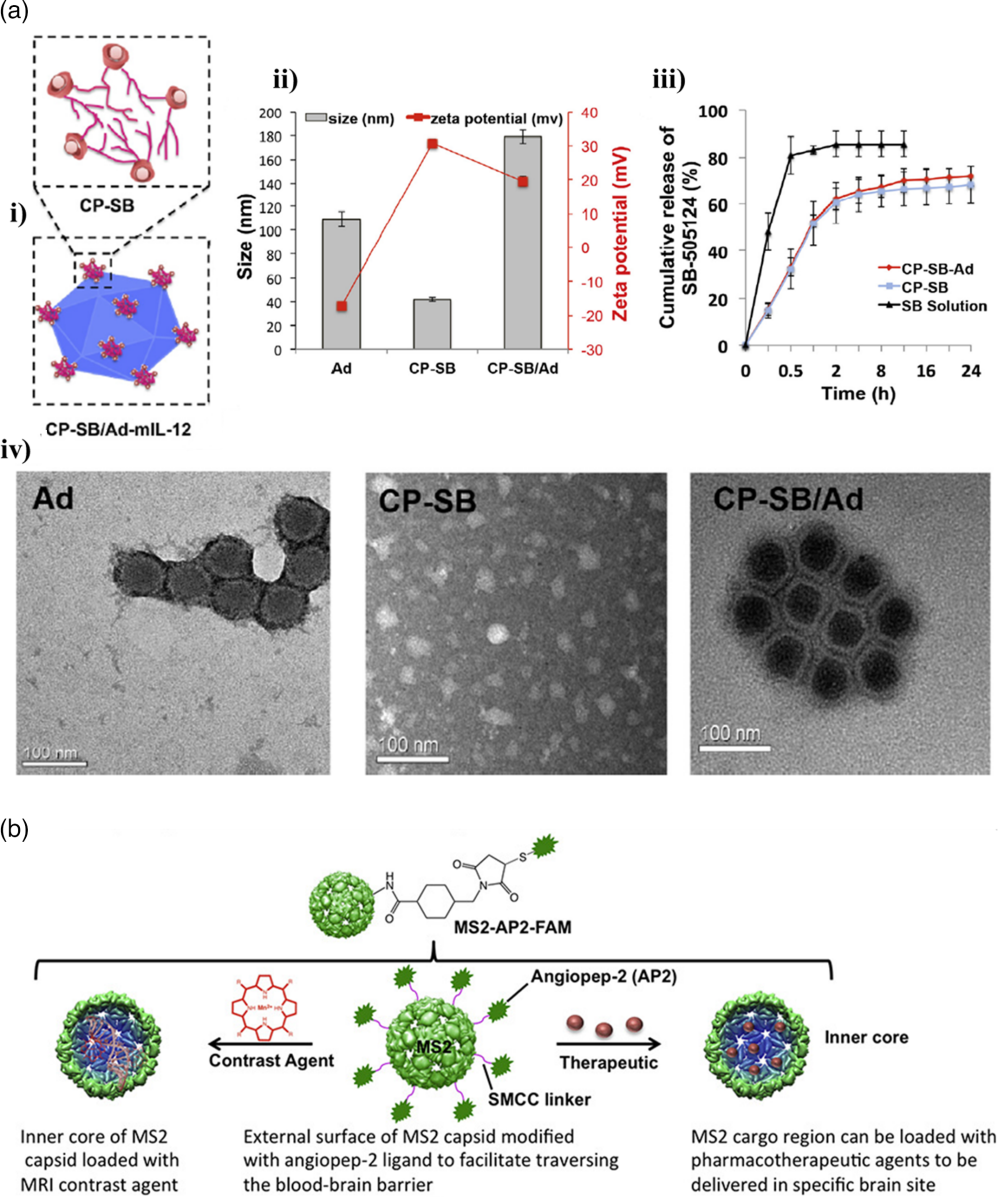
a) CP‐SB/Ad‐mIL‐12 nanocarrier: i) structure's scheme, ii) size and zeta potential measurements of Ad, CP‐Sb complex, and Cp‐SB/Ad, iii) SB release profiles from CP–SB/Ad and CP‐SB, and iv) TEM images of Ad (left), CP‐SB (middle), and CP‐SB/Ad (right). Reproduced with permission.^[^ ^90^ ^]^ Copyright 2017, Elsevier Ltd. b) MS2 bacteriophage carrier system and different applications. Reproduced with permission.^[^ ^91^ ^]^ Copyright 2018, Elsevier Ltd.

Jiang et al.^[^ ^90^ ^]^ created a human adenovirus type 5 (Ad)‐based carrier system capable of delivering interleukin‐12 (IL‐12) and a TGF‐β inhibitor (SB) in order to exert antitumor activity by inducing IFN‐γ secretion, promoting NK and T‐cell proliferation and inhibiting TGF‐β signaling. To achieve this, the group complexed SB with a β‐cyclodextrin‐PEI2k (CP) polymer by adding SB dissolved in DMSO to an aqueous solution of CP at a CP/SB ratio of 1:10. Dialyzing the sample against deionized water allowed the removal of DMSO and free SB leaving the complex CP‐SB in solution. Around 48% of SB was incorporated in the CP‐SB complex. The resulting positively‐charged complex was then attached to the negatively‐charged adenovirus encoding mIL–12 (Ad–mIL–12) through electrostatic interactions (final structure CP‐SB/Ad represented in **Figure** **13**a‐i, zeta potential measurement in Figure 13a‐ii, and TEM images shown in Figure 13a‐iv). DLS experiments revealed that Ad had a mean diameter around 100 nm, CP‐SB complex around 40 nm, and the CP‐SB/Ad carrier around 180 nm (Figure 13a‐ii). The group studied the release profile of SB from the carrier (Figure 13a‐iii) and showed an accelerated release of drug in the first 2 h and a total of SB released around 70% over 24 h.

##### Bacteriophage MS2

The MS2 bacteriophage is an icosahedral virus that only infects bacteria. Its proteinaceous shell is composed of 180 polypeptide chains arranged into 60 triangular monomer units. This viral capsid has a diameter of 28 nm and a 4 nm thickness shell. Contrary to many other viruses, the self‐assembly mechanism is mainly controlled by dimer–dimer contacts of the polypeptide chains.^[^ ^73^ ^]^

Apawu et al.^[^ ^91^ ^]^ developed a carrier system based on the MS2 bacteriophage cage designed to cross the blood–brain barrier (BBB) carrying an MRI contrast agent. The outer surface of the MS2 cage was functionalized with angiopep‐2 (AP2) that has shown promising results in facilitating the transport of molecules across the BBB (see Figure 13b). The MS2 capsid was fabricated by infecting a colony of C3000 *E.* *coli* with plaque‐forming units of MS2 phage and letting it propagate. To functionalize the MS2 surface, the purified bacteriophage was first reacted with succinimidyl 4‐(*N*‐maleimidomethyl)cyclohexane‐1‐carboxylate (SMCC) with a 20% coupling efficiency between available lysine residues on the MS2 and the SMCC. After purification, the MS2‐SMCC conjugate was reacted with the angiopep‐2 with a conjugation efficiency of 70%. The MRI contrast agent Mn^2+^ linked to a porphyrin ring was then encapsulated. The average size of the capsids was 30 nm, each capsid possessed 250 porphyrin rings, and each ring contained one Mn^2+^.

## 4 Artificial Polypeptide‐Based Simple Coacervates

**Figure 14:**
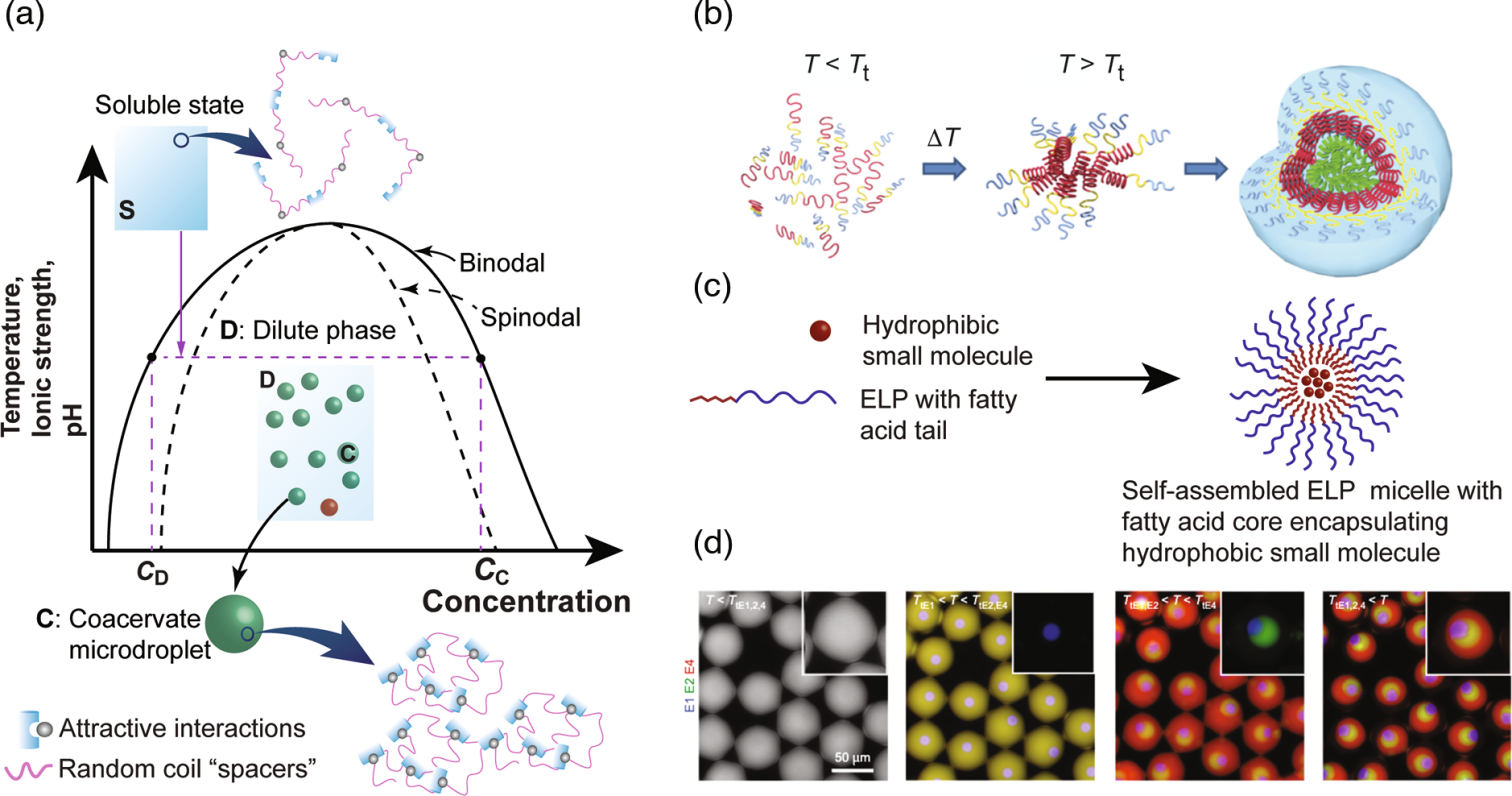
Single‐component coacervation of proteins and polypeptides. a) Schematic phase diagram of the liquid–liquid phase separation process. From the initial single‐phase solution, a change in temperature, ionic strength, or pH leads to a two‐phase system consisting of concentrated microdroplets (coacervates) and a dilute phase. Adapted with permission.^[92d]^ Copyright 2020, The Authors, published on behalf of Materials Research Society by Cambridge University Press. b) Copolymers of ELPs containing both hydrophobic (red) and hydrophilic (blue) domains linked by a Cys‐rich linker (yellow) region. Above the phase transition temperature *T*_t_, the ELPs self‐assemble into nanostructured micelles stabilized by disulfide bonds. Reproduced with permission.^[95b]^ Copyright 2015, Wiley‐VCH. c) ELP with a myristic fatty acid tail genetically incorporated during expression. Above *T*_t_ in the presence of the hydrophobic drug DOx, the ELP/fatty acid conjugate self‐assembles into micelles around a DOx core. Adapted with permission.^[^ ^102^ ^]^ Copyright 2017, Wiley‐VCH. d) Coacervate microdroplets made of mixture of three miscible ELPs with different *T*_t_, each of them labeled with a distinct fluorescent label. As the temperature increases, triple‐layered coacervates are formed, with each layer induced above the specific *T*_t_ of one of the constitutive ELP. Reproduced with permission.^[^ ^103^ ^]^ Copyright 2017, Springer Nature.

Coacervation consists in a LLPS process where fully solvated macromolecules are desolvated upon addition of a salt, an electrolyte compound, a nonsolvent, and/or after a temperature or a pH change, resulting in a two‐phase system (**Figure** **14**a) consisting of concentrated microdroplets in thermodynamic or metastable equilibrium with the depleted solution.^[^ ^92^ ^]^ The polymer‐rich phase is named coacervate whereas the polymer‐depleted phase is called the equilibrium phase, mainly composed of solvent (this terminology has led to some confusion because the coacervate phase can be either in metastable state or in thermodynamic equilibrium with the polymer‐depleted phase depending on the system and specific conditions.).

Perhaps the best‐known example of a simple coacervation protein is human tropoelastin and its mimetic artificial versions, namely, elastin‐like polypeptides (ELPs). Tropoelastin contains both hydrophilic and hydrophobic domains, with the latter characterized by multiple repeats of the pentapeptide [Val‐Gly‐Val‐X‐Pro or VGVXP], where X is often Ala.^[^ ^93^ ^]^ The repetitive regions control coacervation^[^ ^94^ ^]^ via a lower‐critical solution temperature (LCST) mechanism, whereby phase separation occurs above a critical temperature *T*_t_. Comprehensive reviews on tropoelastin biochemistry and biophysical properties have been covered elsewhere.^[^ ^95^ ^]^

Seeking to exploit the LCST properties of tropoelastin, researchers have designed a wide range of artificial ELPs via recombinant technology that can self‐assemble into stimuli‐responsive nanoparticles, micelles, or microcapsules with applications in the biomedical and nanomedicine fields.^[^ ^96^ ^]^ The central idea behind these efforts is to design polypeptides containing multiple copies of the canonical pentapeptide [VGVXP], where X is the guest residue that can be any amino acid except Pro. By varying the number of repeats, the type of guest residues and their molar ratios, copolymers with highly tunable transition temperature *T*_t_ can be obtained.^[^ ^97^ ^]^ Early efforts to obtain micellar‐like structures from ELPs were conducted in 2000 by Conticello and co‐workers,^[^ ^98^ ^]^ who prepared diblock copolymers containing both hydrophilic (with Ala as the guest residue) and hydrophobic (with Val as the guest residue) domains, as well as linker domains with the ionizable Glu residue to enhance interactions with the aqueous solvent. Owing to their amphiphilic characteristics, these ELPs block copolymers assembled into unimodal micelles above the transition temperature with mean diameters of 87, 47, or 57 nm, depending on pH.

The Chilkoti group later expanded these ELP block copolymers by varying the ratio of hydrophobic‐to‐hydrophilic blocks in order to trigger the self‐assembly of micellar structures in the temperature range 37−42 ºC, which is clinically relevant for drug delivery applications. Their design also included cell adhesion peptide motifs such as RGD or NGR to enhance cell internalization.^[^ ^99^ ^]^ In subsequent developments,^[^ ^100^ ^]^ the same group constructed a 160 pentapeptide repeat ELP and added a Cys‐rich domain at the C‐terminus to covalently conjugate the hydrophobic drug doxorubicin (Dox) through the pH‐responsive hydrazone bond. This design enabled the self‐assembly of micellar‐like structures consisting of a Dox‐rich, hydrophobic core surrounded by hydrophilic ELPs. The micelles were internalized by the tumor cells by endocytosis, and owing to the pH‐dependent cleavage of the hydrazone bond, free Dox could be released in the acidic environment of the endosome. Using an analogous design, Kim et al.^[^ ^101^ ^]^ prepared a diblock ELP in which the hydrophobic and hydrophilic domains were separated by a Cys‐rich linker domain. Micelles with monodisperse sizes in the range 25−30 nm were achieved and their assembly was enhanced by incorporating Glu and Tyr residues in the hydrophilic and hydrophobic blocks, respectively, whereas the Cys‐rich linker domains stabilized the micelles and prevented their aggregation by forming disulfide bonds at the core–shell interface (Figure 14b). In another approach to obtain ELP‐based micelles with a hydrophobic drug core, Luginbuhl et al. genetically anchored the fatty myristic acid at the *N*‐terminus (Figure 14c). Varying the length of the ELP regions, they achieved uniform and tunable sizes of micelles in the 20−80 nm range. Furthermore, the use of the myristic fatty acid in the polymer construct allowed physical entrapment of hydrophobic Dox as opposed to covalent immobilization.^[^ ^102^ ^]^ In more recent developments, Simon et al.^[^ ^103^ ^]^ prepared and mixed multiple ELPs with a wide range of hydrophobicity and molecular weights that exhibited transition temperatures varying from 25 up to 90 ºC. These mixtures were then incorporated in w/o emulsion droplets. As the temperature increased, multiple phase separations were triggered within the droplets, each of them governed by the transition temperature of a given ELP. As a result, a remarkable range of hierarchical structures could be generated, including multilayered coacervates (Figure 14d) as well as monodisperse droplets with tunable sizes from ≈50 nm up to 17 µm.

ELP nanoparticles with a simple sequence design (i.e., only one type of guest residue in the sequence) have also been reported in other drug delivery applications; notably, [VPAGV]_220_ has been used to encapsulate and release bone morphogenic growth factor in order to promote bone mineralization.^[104a]^ More recently, the artificial insect protein resilin has also been shown to exhibit a LCST behavior reminiscent of ELPs,^[^ ^105^ ^]^ and resilin‐like polypeptides (RLPs) self‐assembling into nanoparticles by thermal cycling have also been reported.^[^ ^106^ ^]^ These studies suggest that RLPs exhibit a similar potential as ELPs for nano‐ and microcarriers.

Beyond the well‐established ELPs, other types of short peptide‐based coacervates have more recently been discovered and developed.^[104b]^ One class of such peptides investigated by our team are histidine (His)‐rich peptides (HBP*peps*) designed from His‐rich squid beak proteins (HBPs).^[^ ^107^ ^]^ These proteins are characterized by an elevated His content but are otherwise free of other charged residues, resulting in pH‐responsive LLPS slightly below neutral pH. Owing to the pKa of His of ≈6.5, HBPs are in the single‐phase regime in weakly acidic pH but phase‐separate into coacervates above pH 6.5,^[^ ^108^ ^]^ with a rheological response that is strongly sequence‐dependent.^[^ ^109^ ^]^ Similar to ELPs, HB*peps* are modular polypeptides consisting of multiple copies of the pentapeptides Gly‐His‐Gly‐X_1_Y (GHGX_1_Y, where X_1_ is usually the residue Leu, Val, or Pro), Gly‐His‐Gly‐Leu‐His (GHGLH), and Gly‐Ala‐Gly‐Phe‐Ala (GAGFA) that are arranged in multiple permutations. Using solution NMR, Gabryelcyk et al.^[^ ^110^ ^]^ showed that phase separation is a multistep process first triggered by H‐bonding interactions between His and Tyr residues of the GHGLY pentapeptide motif as the pH increases, followed by further stabilization through Tyr–Tyr *π*–*π* stacking. Further studies subsequently revealed that phase separation is driven by specific His and Tyr residues located near the sequence termini, which may result in the formation of well‐defined topological network infinitely expanding into microdroplets.^[^ ^111^ ^]^

**Figure 15:**
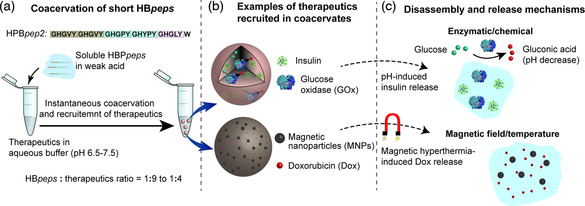
Coacervation of short peptide HB*peps* with concomitant recruitment of therapeutics in the coacervate microdroplets. a) Coacervation of HB*pep2* is induced by simply mixing the soluble peptide within the buffer containing the therapeutics molecules, which are recruited within the droplets during the process. b) Examples of coacervates loaded with insulin and glucose oxidase (GOx, top), and doxorubicin (Dox) and iron oxide magnetic nanoparticles (MNPs, bottom). c) Disassembly and release of therapeutics by various external stimuli. In the case of insulin‐ and GOx‐loaded coacervates, the release is triggered by the conversion of glucose into gluconic acid by GOx that acidifies the interior of the droplets, resulting in pH‐induced disassembly and release. For DOx‐ and MNP‐loaded coacervates, disassembly and release of DOx is triggered by application of an alternative current magnetic field, which increased the temperature within the droplets by magnetic hyperthermia. Figure modified from ref. [112] with permission from the American Chemical Society and Elsevier Ltd., respectively.

A main benefit of pH‐responsive peptide coacervates lays in the ability to recruit client cargos within the microdroplets during the coacervation process, which is readily done by pipetting the peptide solution into a buffer solution containing the cargo of interest (**Figure** **15**a). This one‐pot process occurs instantaneously, under aqueous conditions, and typically with more than 95% recruitment efficiency. Furthermore, disassembly of the coacervates and release of the therapeutic cargo can be activated by external stimuli. All together, these characteristics offer exciting possibilities for controlled release of therapeutics. For example, Lim et al. co‐recruited insulin and GOx within HB*pep*2 microdroplets (Figure 15b).^[112a]^ Upon exposure to glucose, the latter was converted into gluconic acid by GOx (Figure 15c), resulting in localized acidification and disassembly and release of insulin. In this manner, HB*pep*2 coacervates mimicked pancreatic β‐cell function by releasing insulin according to glucose levels. In another proof‐of‐concept study, the hydrophobic drug Dox as well as iron oxide magnetic nanoparticles (MNPs) were recruited within HB*pep*2 droplets (Figure 15b).^[112b]^ Subjecting the loaded droplets to an alternate magnetic field (AMF) resulted in localized heating thanks to the magnetic hyperthermia effect of MNPs, which disassembled the droplets to release Dox (Figure 15c). As loaded HB*pep* coacervates were readily internalized by the liver cancerous cells HepG2, Dox was released directly in the cytosol, thereby killing the cells. These recent results offer interesting perspectives for coacervate microdroplets in nanomedicine and as an efficient transfection agent. Indeed, very recent results have demonstrated that HB*peps* can be conjugated with a redox‐responsive moiety that is responsive to the cell‐endogenous peptide glutathione, such that the microdroplets can be disassembled intracellularly in a broad range of cells to efficiently release a wide range of therapeutic payloads, including proteins, anticancer peptides, and mRNAs.^[^ ^113^ ^]^

The lab of Spruijt has recently reported coacervate microdroplets made of even smaller peptidic‐based units.^[^ ^114^ ^]^ They designed dipeptides from the hydrophobic amino acids phenylalanine (Phe) and leucine (Leu), which they joined together through the hydrophilic, disulfide‐containing cystamine linker. The formation of micrometer size droplets was achieved by shifting the pH from 5−6 to 7. During the phase separation process, small molecules such as fluorophores and nucleic acids could be sequestrated within the droplets. Owing to the presence of the disulfide linker, disassembly and reformation of the coacervates could be controlled by the disulfide/thiol redox chemistry.

Given the exponential growth of the LLPS field in recent years that has resulted in a large number of both intracellular and extracellular proteins exhibiting coacervation/LLPS, it is very likely that these discoveries can be exploited to engineer micro‐ and nano‐carriers.^[^ ^115^ ^]^ As an illustrative example, Hughes et al. have identified a series of low‐complexity aromatic‐rich kink segments (LARKS) from eukaryotic proteomes that assemble into reversible oligomers stabilized by steric zippers. Such short peptides are prime candidates to create artificial protocells with highly tunable stimuli‐responsive characteristics.^[^ ^116^ ^]^

## 5 Conclusion

Thanks to their specific properties, considerable efforts have been paid to the preparation of micro‐ and nano‐capsules made of protein shells. Most of the capsules are designed to be used in applications directly connected with humans: drug delivery, bioimaging, and conservation of food and drugs. Capsule size is a parameter of extreme importance depending on the type of targeted application. Microcapsules are interesting for food‐related applications and imaging in arteries or blood vessels that are large enough to let them flow through. Nanocapsules find applications in drug delivery and bioimaging, where crossing biological barriers and small blood vessels with slow blood circulation is sought.

Other parameters such as size dispersity of the capsules, cost, and efficiency of production also have an impact on the final application. The different techniques discussed in this review allow the fabrication of capsule samples with different properties; therefore, choosing the appropriate technique is essential for the desired application. As an example, spray drying is used when large quantities need to be cheaply produced without considering size distribution, e.g., to encapsulate food. In contrast, microfluidic techniques have a lower production output and require a bigger investment, but they allow a precise control over size dispersity which is critical in biomedical applications.

Choosing the right protein for the right application is also very important. Plant‐based proteins such as WPI and SPI are mainly used for conservation of substances or encapsulation of food because there are concerns for biomedical applications due to their immunogenic properties. Human‐based proteins and polypeptides such as Hb, HSA, or ELPs are the best choices for drug delivery, as well as carriers based on short peptides given the low immunogenicity of the latter. To improve stability and circulation time of the capsules in serum conditions, synthetic polymers such as PEG are often anchored to the surface of the capsules. Small peptides (such as cell‐penetrating peptides) and antibodies are also grafted to the surface of the objects to increase tumor specificity and internalization.

In recent years, scientists started to produce replicas of protein cages that already exist in nature to use them as carriers for bioimaging and drug and gene delivery applications. These types of capsules, which are now routinely produced through recombinant technology, possess very well‐defined sizes and structure, which is of great interest for the targeted applications. Finally, the small number of publications on protein shell nanocapsules shows that generating these kinds of objects remains a challenge for the scientific community.

While the evolution of capsules as carriers for therapeutics has evolved in the last few decades, there remain challenges to be overcome. Reducing formulation toxicity and immunological response while increasing disease‐ and cell‐specificity are essential to improve treatment efficacy. Encapsulation and release of the cargo need to be fully controlled to maintain the physicochemical and biological activities of the encapsulated molecules. The protocols for capsule generation and storage need to be simple and efficient enough to allow future scale‐up. Long‐term performance and safety of these type of materials also need to be carefully assessed. Continuous need for new, more reliable, and effective technologies for drug delivery will surely increase the investment in nanotechnology‐based protein carriers and make way to solving the existing problems.

## Conflict of Interest

The authors declare no conflict of interest.

## References

[1] F. Podczeck , B. Jones , Pharmaceutical Capsules, Pharmaceutical Press, London, Chicago 2004.

[2] L. J. De Cock , S. De Koker , B. G. De Geest , J. Grooten , C. Vervaet , J. P. Remon , G. B. Sukhorukov , M. N. Antipina , Angew. Chem. Int. Ed. 2010, 49, 6954. 10.1002/anie.200906266 20645362

[3] F. Casanova , L. Santos , J. Microencapsul. 2016, 33, 1. 26612271 10.3109/02652048.2015.1115900

[4] B. Peña , C. Panisello , G. Aresté , R. Garcia-Valls , T. Gumí , Chem. Eng. J. 2012, 179, 394.

[5] M. Schoebitz , M. D. López , A. Roldán , Agron. Sustain. Dev. 2013, 33, 751.

[6] A. C. Sather , H. G. Lee , J. R. Colombe , A. Zhang , S. L. Buchwald , Nature 2015, 524, 208. 26268191 10.1038/nature14654 PMC4536573

[7] E. Amstad , ACS Macro Lett. 2017, 6, 841.

[8] C. E. Mora-Huertas , H. Fessi , A. Elaissari , Int. J. Pharm. 2010, 385, 113. 19825408 10.1016/j.ijpharm.2009.10.018

[9] M. Vert , Y. Doi , K-H. Hellwich , M. Hess , P. Hodge , P. Kubisa , M. Rinaudo , F. Schué , Appl. Pure Chem. 2012, 84, 377.

[10] L. L. Balassa , G. O. Fanger , O. B. Wurzburg , C R C Crit. Rev. Food Technol. 1971, 2, 245.

[11] L. Sánchez-Silva , J. F. Rodríguez , A. Romero , P. Sánchez , J. Appl. Polym. Sci. 2011, 124, 4809.

[12] M. José Alonso , Biomed. Pharmacother. 2004, 58, 168.15082339

[13] a) P. Couvreur , G. Barratt , E. Fattal , C. Vauthier , Crit. Rev. Ther. Drug Carrier Syst. 2002, 19, 99; 12197610 10.1615/critrevtherdrugcarriersyst.v19.i2.10

[14] J. Lademann , A. Patzelt , H. Richter , O. Lademann , G. Baier , L. Breucker , K. Landfester , Laser Phys. Lett. 2013, 10, 083001.

[15] L.Y. Zhang , Y. Wang , Y. Cao , D. Lou , B. Wang , J. Biomed. Mater. Res. A 2013, 101 3661. 23983207 10.1002/jbm.a.34635

[16] C. I. Branden , J. Tooze , Introduction To Protein Structure, Garland Science, New York, NY 2012.

[17] F. B. Jensen , A. Fago , R. E. Weber , in Fish Physiology (Eds: S. F. Perry , B. L. Tufts ), Vol. 17, Academic Press, New York, NY, 1998, pp. 1–32.

[18] D. Whitford , in Proteins: Structure And Function, John Wiley & Sons, Hoboken, NJ 2013.

[19] S. Nagarajan , S. Radhakrishnan , S. N. Kalkura , S. Balme , P. Miele , M. Bechelany , Macromol. Chem. Phys. 2019, 220, 1900126.

[20] J. E. Gagner , W. Kim , E. L. Chaikof , Acta Biomater. 2004, 10, 1542. 10.1016/j.actbio.2013.10.001 PMC3960372 24121196

[21] U. Shimanovich , G. J. L. Bernardes , T. P. J. Knowles , A. Cavaco-Paulo , Rev. Chem. Soc. 2014, 43, 1361. 10.1039/c3cs60376h 24336689

[22] A. Gedanken , Chem. Eur. J. 2008, 14, 3840. 18306264 10.1002/chem.200701541

[23] a) N. Aggarwal , D. Eliaz , H. Cohen , I. Rosenhek-Goldian , S. R. Cohen , A. Kozell , T. O. Mason , U. Shimanovich , Commun. Chem. 2021, 4, 1; 36697777 10.1038/s42004-021-00494-2 PMC9814780

[24] J. C. Colmenares , G. Chatel , in Sonochemistry: From Basic Principles To Innovative Applications, Springer, New York 2017. 10.1007/s41061-016-0096-1 28044279

[25] M. W. Grinstaff , K. S. Suslick Proc. Natl. Acad. Sci. 1991, 88, 7708. 1652761 10.1073/pnas.88.17.7708 PMC52371

[26] S. Avivi , A. Gedanken , Biochem. J. 2002, 366, 705. 12119047 10.1042/BJ20020676 PMC1222846

[27] S. Unnikrishnan , A. L. Klibanov , Am. J. Roentgenol. 2012, 199, 292. 22826389 10.2214/AJR.12.8826

[28] a) J. C. McDonald , D. C. Duffy , J. R. Anderson , D. T. Chiu , H. Wu , O. J. A. Schueller , G. M. Whitesides , Electrophoresis 2000, 21, 27; 10634468 10.1002/(SICI)1522-2683(20000101)21:1<27::AID-ELPS27>3.0.CO;2-C

[29] J. I. Park , D. Jagadeesan , R. Williams , W. Oakden , S. Chung , G. J. Stanisz , ACS Nano 2010, 4, 6579. 20968309 10.1021/nn102248g

[30] M. Seo , I. Gorelikov , R. Williams , N. Matsuura , Langmuir 2010, 26, 13855. 20666507 10.1021/la102272d

[31] J. I. Park , Z. Nie , A. Kumachev , A. I. Abdelrahman , B. P. Binks , H. A. Stone , E. Kumacheva , Angew. Chem. Int. Ed. 2009, 48, 5300. 10.1002/anie.200805204 19301346

[32] F. E. Angilè , K. B. Vargo , C. M. Sehgal , D. A. Hammer , D. Lee , Langmuir 2014, 30, 12610. 25265041 10.1021/la502610c PMC4211726

[33] X-L. Gu , X. Zhu , X-Z. Kong , Y. Tan , J. Microencapsul. 2010, 27, 355. 20163286 10.3109/02652040903221532

[34] D. H. Everett , Pure Appl. Chem. 1972, 31, 577.

[35] A. Yaqoob Khan , S. Talegaonkar , Z. Iqbal , F. J. Ahmed , R. K. Khar , Curr. Drug Deliv. 2006, 3 429. 17076645 10.2174/156720106778559056

[36] K. S. Suslick , M. W. Grinstaff , J. Am. Chem. Soc. 1990, 112, 7807.

[37] Z. Li , F. Xu , Q. Li , S. Liu , H. Wang , H. Möhwald , X. Cui , Colloids Surf. B 2015, 136, 470. 10.1016/j.colsurfb.2015.09.056 26454053

[38] M. C. Mauguet , J. Legrand , L. Brujes , G. Carnelle , C. Larre , Y. Popineau , J. Microencapsul. 2002, 19, 377. 12022503 10.1080/02652040110105346

[39] J. Lazko , Y. Popineau , J. Legrand , Colloids Surf. B 2004, 37, 1. 10.1016/j.colsurfb.2004.06.004 15450301

[40] Y. P. Timilsena , T. O. Akanbi , N. Khalid , B. Adhikari , C. J. Barrow , Int. J. Biol. Macromol. 2019, 121, 1276. 30352231 10.1016/j.ijbiomac.2018.10.144

[41] B. K. Green , US2800458A, 1957.

[42] a) R. Dubey , T. C. Shami , K. U. B. Rao , Def. Sci. J. 2009, 59 14;

[43] a) L. A. Luzzi , R. J. Gerraughty , J. Pharm. Sci. 1964, 53, 429; 14189940 10.1002/jps.2600530419

[44] a) H. Jizomoto , E. Kanaoka , K. Sugita , K. Hirano , Pharm. Res. 1993, 10, 1115. 8415395 10.1023/a:1018951814939

[45] S. S. Deveci , G. Basal , Colloid Polym. Sci. 2009, 287, 1455.

[46] a) N. Eghbal , R. Choudhary , LWT 2018, 90, 254;

[47] A. C. Schloss , W. Liu , D. M. Williams , G. Kaufman , H. P. Hendrickson , B. Rudshteyn , L. Fu , H. Wang , V. S. Batista , C. Osuji , E. C. Y. Yan , L. Regan , ACS Biomater. Sci. Eng. 2016, 2, 1856. 29805990 10.1021/acsbiomaterials.6b00447 PMC5967246

[48] G. Kaufman , W. Liu , D. M. Williams , Y. Choo , M. Gopinadhan , N. Samudrala , R. Sarfati , E. C. Y. Yan , L. Regan , C. O. Osuji , Langmuir 2017, 33, 13590. 29094950 10.1021/acs.langmuir.7b03226

[49] a) U. Shimanovich , F. S. Ruggeri , E. De Genst , J. Adamcik , T. P. Barros , D. Porter , T. Müller , R. Mezzenga , C. M. Dobson , F. Vollrath , C. Holland , T. P. J. Knowles , Nat. Commun. 2017, 8, 15902; 28722016 10.1038/ncomms15902 PMC5524934

[50] C. Anandharamakrishnan , S. P. Ishwarya , in Spray Drying Techniques For Food Ingredient Encapsulation, Wiley, Chichester, UK 2015.

[51] Y. D. Kim , C. V. Morr , J. Agric. Food Chem. 1996, 44, 1314.

[52] R. Wang , Z. Tian , L. Chen , Food Res. Int. 2011, 44, 2735.

[53] C. Arpagaus , A. Collenberg , D. Rütti , E. Assadpour , S. M. Jafari , Int. J. Pharm. 2018, 546, 194. 29778825 10.1016/j.ijpharm.2018.05.037

[54] X. Li , N. Anton , C. Arpagaus , F. Belleteix , T. F. Vandamme , J. Control. Release 2010, 147, 304. 20659510 10.1016/j.jconrel.2010.07.113

[55] A. Nesterenko , I. Alric , F. Silvestre , V. Durrieu , Ind. Crops Prod. 2013, 42, 469.

[56] a) A. P. T. R. Pierucci , L. R. Andrade , M. Farina , C. Pedrosa , M. H. M. Rocha-Leão , J. Microencapsul. 2007, 24, 201; 17454432 10.1080/02652040701281167

[57] G. F. Decher , Science 1997, 277, 1232.

[58] a) F. J-J. Toublan , S. Boppart , K. S. Suslick , J. Am. Chem. Soc. 2006, 128, 3472; 16536492 10.1021/ja0544455

[59] W. Tong , X. Song , C. Gao , Chem. Soc. Rev. 2012, 41, 6103. 22695830 10.1039/c2cs35088b

[60] L. Duan , Q. He , X. Yan , Y. Cui , K. Wang , J. Li , Biochem. Biophys. Res. Commun. 2007, 354, 357. 17241614 10.1016/j.bbrc.2006.12.223

[61] L. Duan , W. Qi , X. Yan , Q. He , Y. Cui , K. Wang , D. Li , J. Li , J. Phys. Chem. B 2009, 113, 395. 19090667 10.1021/jp807883e

[62] W. Qi , X. Yan , L. Duan , Y. Cui , Y. Yang , J. Li , Biomacromolecules 2009, 10, 1212. 19323511 10.1021/bm801502r

[63] a) L. Duan , X. Yan , A. Wang , Y. Jia , J. Li , ACS Nano 2012, 6, 6897; 22732258 10.1021/nn301735u

[64] Y. Zhu , W. Tong , C. Gao , Soft Matter 2011, 7, 5805.

[65] O. Shchepelina , I. Drachuk , M. K. Gupta , J. Lin , V. V. Tsukruk , Adv. Mater. 2011, 23, 4655. 21915919 10.1002/adma.201102234

[66] D. Mertz , J. Cui , Y. Yan , G. Devlin , C. Chaubaroux , A. Dochter , R. Alles , P. Lavalle , J. C. Voegel , A. Blencowe , P. Auffinger , F. Caruso , ACS Nano 2012, 6, 7584. 22950440 10.1021/nn302024t

[67] Z. An , G. Lu , H. Möhwald , J. Li , Chem. Eur. J. 2004, 10, 5848. 15481026 10.1002/chem.200400090

[68] a) Z. An , C. Tao , G. Lu , H. Möhwald , S. Zheng , Y. Cui , J. Li , Chem. Mater. 2005, 17, 2514;

[69] C. Ye , O. Shchepelina , R. Calabrese , I. Drachuk , D. L. Kaplan , V. V. Tsukruk , Biomacromolecules 2011, 12, 4319. 22050007 10.1021/bm201246f PMC3404390

[70] M. A. Serban , D. L. Kaplan , Biomacromolecules 2010, 11, 3406. 21028849 10.1021/bm100925s PMC3005850

[71] C. Ye , I. Drachuk , R. Calabrese , H. Dai , D. L. Kaplan , V. V. Tsukruk , Langmuir 2012, 28, 12235. 22834790 10.1021/la302455y

[72] K. Piradashvili , M. Fichter , K. Mohr , S. Gehring , F. R. Wurm , K. Landfester , Biomacromolecules 2015, 16, 815. 25619361 10.1021/bm5016915

[73] M. Rother , M. G. Nussbaumer , K. Renggli , N. Bruns , Chem. Soc. Rev. 2016, 45, 6213. 27426103 10.1039/c6cs00177g

[74] Y. Yang , Y. Jia , L. Gao , J. Fei , L. Dai , J. Zhao , J. Li , Chem. Commun. 2011, 47, 12167. 10.1039/c1cc16004d 22005711

[75] X. Wang , D. Miao , X. Liang , J. Liang , C. Zhang , J. Yang , D. Kong , C. Wang , H. Sun , Biomater. Sci. 2017, 5, 658. 28246671 10.1039/c6bm00915h

[76] a) C. Lee , A. Hwang , L. Jose , J. H. Park , J. K. Song , K. Shim , S. S. A. An , H. Paik , Biomacromolecules 2018, 19, 4219; 30265806 10.1021/acs.biomac.8b00965

[77] Y. Lv , F. Yang , X. Li , X. Zhang , S. Abbas , Food Hydrocoll. 2014, 35, 305.

[78] W. He , Y. Lu , J. Qi , L. Chen , F. Hu , W. Wu , Int. J. Pharm. 2013, 445, 69. 23396257 10.1016/j.ijpharm.2013.01.072

[79] a) M. Fichter , K. Piradashvili , A. Pietrzak-Nguyen , L. Pretsch , G. Kuhn , S. Strand , M. Knuf , F. Zepp , F. R. Wurm , V. Mailänder , K. Landfester , S. Gehring , Biomaterials 2016, 108, 1; 27614817 10.1016/j.biomaterials.2016.08.046

[80] X. Yan , M. Delgado , A. Fu , P. Alcouffe , S. G. Gouin , E. Fleury , J. L. Katz , F. Ganachaud , J. Bernard , Angew. Chem. Int. Ed. 2014, 53, 6910. 10.1002/anie.201402825 24862553

[81] a) X. Yan , R. Ramos , E. Hoibian , C. Soulage , P. Alcouffe , F. Ganachaud , J. Bernard , ACS Macro Lett. 2017, 6, 447; 35610850 10.1021/acsmacrolett.7b00094

[82] R. Ramos , K. Koh , B. Gabryelczyk , L. Chai , D. Kanagavel , X. Yan , F. Ganachaud , A. Miserez , J. Bernard , ACS Macro Lett. 2021, 10, 628. 35570771 10.1021/acsmacrolett.1c00171

[83] A. Miserez , J. C. Weaver , P. B. Pedersen , T. Schneeberk , R. T. Hanlon , D. Kisailus , H. Birkedal , Adv. Mater. 2009, 21, 401.

[84] a) P. A. Guerette , S. Hoon , D. Ding , S. Amini , A. Masic , V. Ravi , B. Venkatesh , J. C. Weaver , A. Miserez , ACS Nano 2014, 8, 7170; 24911543 10.1021/nn502149u

[85] N. M. Molino , S-W. Wang , Curr. Opin. Biotechnol. 2014, 28, 75. 24832078 10.1016/j.copbio.2013.12.007 PMC4087095

[86] X. Guan , Y. Chang , J. Sun , J. Song , Y. Xie , Macromol. Biosci. 2018, 18, 1800013. 10.1002/mabi.201800013 29665276

[87] F. Palombarini , E. Di Fabio , A. Boffi , A. Macone , A. Bonamore , Molecules 2020, 25, 825. 32070033 10.3390/molecules25040825 PMC7070480

[88] H. Huang , K. Sha , H. Veroniaina , Z. Wu , Z. Wu , X. Qi , Nanoscale 2020, 12, 7347. 32206764 10.1039/d0nr00547a

[89] R. Li , Y. Ma , Y. Dong , Z. Zhao , C. You , S. Huang , X. Li , F. Wang , Y. Zhang , ACS Biomater. Sci. Eng. 2019, 5, 6645. 33423483 10.1021/acsbiomaterials.9b01533

[90] J. Jiang , Y. Zhang , K. Peng , Q. Wang , X. Hong , H. Li , G. Fan , Z. Zhang , T. Gong , X. Sun , Acta Biomater. 2017, 61, 114. 28483693 10.1016/j.actbio.2017.05.009

[91] A. K. Apawu , S. M. Curley , A. R. Dixon , M. Hali , M. Sinan , R. D. Braun , J. Castracane , A. T. Cacace , M. Bergkvist , A. G. Holt , Nanomed. Nanotechnol. Biol. Med. 2018, 14, 1999. 10.1016/j.nano.2018.04.003 29665440

[92] a) C. P. Brangwynne , P. Tompa , R. V. Pappu , Nat. Phys. 2015, 11, 899;

[93] B. Vrhovski , A. S. Weiss , Eur. J. Biochem. 1998, 258, 1. 9851686 10.1046/j.1432-1327.1998.2580001.x

[94] G. C. Yeo , F. W. Keeley , A. S. Weiss , Adv. Colloid Interfaces 2010, 167, 94. 10.1016/j.cis.2010.10.003 21081222

[95] a) S. G. Wise , G. C. Ye , M. A. Hiob , J. Rnjak-Kovacina , D. L. Kapla , M. K. C. Ng , A. S. Weiss , Acta Biomater. 2014, 10, 1532–1541; 23938199 10.1016/j.actbio.2013.08.003 PMC3879170

[96] A. K. Varanko , J. C. Su , A. Chilkoti , Annu. Rev. Biomed. Eng. 2020, 22, 1. 32343908 10.1146/annurev-bioeng-092419-061127

[97] a) D. W. Urry , D. C. Gowda , T. M. Parker , C-H. Luan , M. C. Reid , C. M. Harris , A. Pattanaik , R. Dean Harris , Biopolymers 1992, 32, 1243; 1420991 10.1002/bip.360320913

[98] T. A. T. Lee , A. Cooper , R. P. Apkarian , V. P. Conticello , Adv. Mater. 2000, 12, 1105.

[99] M. R. Dreher , A. J. Simnick , K. Fischer , R. J. Smith , A. Patel , M. Schmidt , A. Chilkoti , J. Am. Chem. Soc. 2008, 130, 687. 18085778 10.1021/ja0764862 PMC2855373

[100] J. A. MacKay , M. Chen , J. R. McDaniel , W. Liu , A. J. Simnick , A. Chilkoti , Nat. Mater. 2009, 8, 993. 19898461 10.1038/nmat2569 PMC2862348

[101] W. Kim , J. Thévenot , E. Ibarboure , S. Lecommandoux , E. L. Chaikof , Angew. Chem. Int. Ed. 2010, 49, 4257. 10.1002/anie.201001356 20446331

[102] K. M. Luginbuhl , D. Mozhdehi , M. Dzuricky , P. Yousefpour , F. C. Huang , N. R. Mayne , K. L. Buehne , A. Chilkoti , Angew. Chem. Int. Ed. 2017, 56, 13979. 10.1002/anie.201704625 PMC5909378 28879687

[103] J. R. Simon , N. J. Carroll , M. Rubinstein , A. Chilkoti , G. P. López , Nat. Chem. 2017, 9, 509. 28537592 10.1038/nchem.2715 PMC5597244

[104] a) P. C. Bessa , R. Machado , S. Nürnberger , D. Dopler , A. Banerjee , A. M. Cunha , J. C. Rodríguez-Cabello , H. Redl , M. van Griensven , R. L. Reis , M. Casal , J. Control. Release 2010, 142, 312; 19913578 10.1016/j.jconrel.2009.11.003

[105] R. S-C. Su , Y. Kim , J. C. Liu , Acta Biomater. 2014, 10, 1601. 23831198 10.1016/j.actbio.2013.06.038

[106] L. Li , T. Luo , K. L. Kiick , Macromol. Rapid Commun. 2015, 36, 90. 25424611 10.1002/marc.201400521 PMC4552326

[107] a) A. Miserez , T. Schneberk , C. Sun , F. W. Zok , J. H. Waite , Science 2008, 319, 1816; 18369144 10.1126/science.1154117 PMC2754134

[108] a) Y. Tan , S. Hoon , P. A. Guerette , W. Wei , A. Ghadban , C. Hao , A. Miserez , J. H. Waite , Nat. Chem. Biol. 2015, 11, 488; 26053298 10.1038/nchembio.1833

[109] H. Cai , B. Gabryelczyk , M. S. S. Manimekalai , G. Grüber , S. Salentinig , A. Miserez , Soft Matter 2017, 13, 7740. 29043368 10.1039/c7sm01352c

[110] B. Gabryelczyk , H. Cai , X. Shi , Y. Sun , P. J. M. Swinkels , S. Salentinig , K. Pervushin , A. Miserez , Nat. Commun. 2019, 10, 5465. 31784535 10.1038/s41467-019-13469-8 PMC6884462

[111] J. Lim , A. Kumar , K. Low , C. S. Verma , Y. Mu , A. Miserez , K. Pervushin , J. Phys. Chem. B 2021, 125, 6776. 34106723 10.1021/acs.jpcb.0c11476

[112] a) Z. W. Lim , Y. Ping , A. Miserez , Bioconjug. Chem. 2018, 29, 2176; 29944344 10.1021/acs.bioconjchem.8b00369

[113] Y. Sun , S. Y. Lau , Z. W. Lim , S. C. Chang , F. Ghadessy , A. Partridge , A. Miserez , 2021, 10.21203/rs.3.rs-181485/v1, accepted for publication in "Nature Chemistry". 35115657

[114] M. Abbas , W. P. Lipiński , K. K. Nakashima , W. T. S. Huck , E. Spruijt , Nat. Chem. 2021, 13, 1046. 34645986 10.1038/s41557-021-00788-x

[115] a) D. Bracha , M. T. Walls , C. P. Brangwynne , Nat. Biotechnol. 2019, 37, 1435; 31792412 10.1038/s41587-019-0341-6

[116] M. P. Hughes , M. R. Sawaya , D. R. Boyer , L. Goldschmidt , J. A. Rodriguez , D. Cascio , L. Chong , T. Gonen , D. S. Eisenberg , Science 2018, 359, 698. 29439243 10.1126/science.aan6398 PMC6192703

[117] X. Jun-Xia , Y. Hai-Yan , Y. Jian , Food Chem. 2011, 125, 1267.

[118] D. V. Mendanha , S. E. Molina Ortiz , C. S. Favaro-Trindade , A. Mauri , E. S. Monterrey-Quintero , M. Thomazini , Food Res. Int. 2009, 42, 1099.

[119] C. Butstraen , F. Salaün , Carbohydr. Polym. 2014, 99, 608. 24274550 10.1016/j.carbpol.2013.09.006

[120] F. Weinbreck , M. Minor , C. G. de Kruif , J. Microencapsul. 2004, 21, 667. 15762323 10.1080/02652040400008499

[121] G. A. Rocha-Selmi , F. T. Bozza , M. Thomazini , H. M. A. Bolini , C. S. Fávaro-Trindade , Food Chem. 2013, 139, 72. 23561080 10.1016/j.foodchem.2013.01.114

[122] M. L. Flenniken , L. O. Liepold , B. E. Crowley , D. A. Willits , M. J. Young , T. Douglas , Chem. Commun. 2005, 4, 447. 10.1039/b413435d 15654365

[123] A. Ma-Ham , H. Wu , J. Wang , X. Kang , Y. Zhang , Y. Lin , J. Mater. Chem. 2011, 21, 8700.

[124] Z. Yang , X. Wang , H. Diao , J. Zhang , H. Li , H. Sun , Z. Guo , Chem. Commun. 2007, 33, 3453. 10.1039/b705326f 17700879

[125] X. Liu , W. Wei , S. Huang , S-S. Lin , X. Zhang , C. Zhang , Y. Du , G. Ma , M. Li , S. Mann , D. Ma , J. Mater. Chem. B 2013, 1, 3136. 32260913 10.1039/c3tb20081g

[126] Z. Zhen , W. Tang , H. Chen , X. Lin , T. Todd , G. Wang , T. Cowger , X. Chen , J. Xie , ACS Nano 2013, 7, 4830. 23718215 10.1021/nn305791q PMC3705644

[127] D. Ren , F. Kratz , S-W. Wang , Small 2011, 7, 1051. 21456086 10.1002/smll.201002242 PMC3118673

[128] N. M. Molino , A. K. L. Anderson , E. L. Nelson , S.-W. Wang , ACS Nano 2013, 7, 9743. 24090491 10.1021/nn403085w PMC3893022

[129] M. Zochowska , A. Paca , G. Schoehn , J-P. Andrieu , J. Chroboczek , B. Dublet , E. Szolajska , PLoS One 2009, 4, 12. 10.1371/journal.pone.0005569 PMC2679213 19440379

[130] L. Shan , S. Cui , C. Du , S. Wan , Z. Qian , S. Achilefu , Y. A Gu , Biomaterials 2012, 33, 146. 21959006 10.1016/j.biomaterials.2011.09.025

[131] A. A. A. Aljabali , S. Shukla , G. P. Lomonossoff , N. F. Steinmetz , D. J. Evans , Mol. Pharm. 2013, 10, 3. 22827473 10.1021/mp3002057 PMC3564650

[132] I. Yildiz , K. L. Lee , K. Chen , S. Shukla , N. F. Steinmetz , J. Control. Release 2013, 172, 568. 23665254 10.1016/j.jconrel.2013.04.023 PMC3815978

[133] C. E. Ashley , E. C. Carnes , G. K. Phillips , P. N. Durfee , M. D. Buley , C. A. Lino , D. P. Padilla , B. Phillips , M. B. Carter , C. L. Willman , C. J. Brinker , J. do C. Caldeira , B. Chackerian , W. Wharton , D. S. Peabody , ACS Nano 2011, 5, 5729. 21615170 10.1021/nn201397z PMC3144304

[134] Y. Pan , Y. Zhang , T. Jia , K. Zhang , J. Li , L. Wang , FEBS J. 2012, 279, 1198. 22309233 10.1111/j.1742-4658.2012.08512.x

[135] J. K. Pokorski , M. L. Hovlid , M. G. Finn , ChemBioChem 2011, 12, 2441. 21956837 10.1002/cbic.201100469 PMC3410710

